# A Novel SNP-STR System Based on a Capillary Electrophoresis Platform

**DOI:** 10.3389/fgene.2021.636821

**Published:** 2021-02-05

**Authors:** Hui Jian, Li Wang, Meili Lv, Yu Tan, Ranran Zhang, Shengqiu Qu, Jijun Wang, Lagabaiyila Zha, Lin Zhang, Weibo Liang

**Affiliations:** ^1^Department of Forensic Genetics, West China School of Basic Medical Sciences and Forensic Medicine, Sichuan University, Chengdu, China; ^2^Department of Obstetrics and Gynecology, West China Second University Hospital, Sichuan University, Chengdu, China; ^3^Key Laboratory of Birth Defects and Related Diseases of Women and Children (Sichuan University), Ministry of Education, Chengdu, China; ^4^Department of Immunology, West China School of Basic Medical Sciences and Forensic Medicine, Sichuan University, Chengdu, China; ^5^HI-TECH Industrial Sub-Branch of Chengdu Municipal Public Security Bureau, Chengdu, China; ^6^Department of Forensic Medicine, School of Basic Medical Sciences, Central South University, Changsha, China

**Keywords:** SNP-STR, microhaplotype, capillary electrophoresis, forensic genetics, unbalanced DNA mixtures, likelihood ratio (LR)

## Abstract

Various compound markers encompassing two or more variants within a small region can be regarded as generalized microhaplotypes. Many of these markers have been investigated for various forensic purposes, such as individual identification, deconvolution of DNA mixtures, or forensic ancestry inference. SNP-STR is a compound biomarker composed of a single nucleotide polymorphism (SNP) and a closely linked short tandem repeat polymorphism (STR), and possess the advantages of both SNPs and STRs. In addition, in conjunction with a polymerase chain reaction (PCR) technique based on the amplification refractory mutation system (ARMS), SNP-STRs can be used for forensic unbalanced DNA mixture analysis based on capillary electrophoresis (CE), which is the most commonly used platform in worldwide forensic laboratories. Our previous research reported 11 SNP-STRs, but few of them are derived from the commonly used STR loci, for which existing STR databases can be used as a reference. For maximum compatibility with existing DNA databases, in this study, we screened 18 SNP-STR loci, of which 14 were derived from the expanded CODIS core loci set. Stable and sensitive SNP-STR multiplex PCR panels based on the CE platform were established. Assays on simulated two-person DNA mixtures showed that all allele-specific primers could detect minor DNA components in 1:500 mixtures. Population data based on 113 unrelated Chengdu Han individuals were investigated. A Bayesian framework was developed for the likelihood ratio (LR) evaluation of SNP-STR profiling results obtained from two-person mixtures. Furthermore, we report on the first use of SNP-STRs in casework to show the advantages and limitations for use in practice. Compared to 2.86 × 10^3^ for autosomal STR kits, the combined LR reached 7.14 × 10^7^ using the SNP-STR method in this casework example.

## Introduction

Microhaplotypes has been revealed the abilities in different forensic application purposes, including individual identification, mixture recognition (Chen et al., 2018; Oldoni and Podini, 2019; Oldoni et al., 2020), and ancestry inference (Bulbul et al., 2018; Chen et al., 2019). In our opinion, compound biomarkers consisting of two or more variants that occur within a small region, can be regarded as generalized microhaplotypes, and include single nucleotide polymorphisms (SNPs) closely linked to short tandem repeats (STRs) (SNP-STR), insertion and deletion polymorphisms (indels) closely linked to STRs (DIP-STR), indel polymorphisms closely linked to SNPs (DIP-SNPs), and several indel polymorphisms physically linked very tightly (multi-indels) (Castella et al., 2013; Wang et al., 2015; Wendt et al., 2016; Tan et al., 2017, 2018; Qu et al., 2020). Among various markers, STRs are the most popular multiallelic markers used in forensics worldwide. Since they are highly polymorphic and discriminative among individuals, they were adopted as reference loci for the Combined DNA Index System (CODIS) and also facilitated the worldwide implementation of the crime National DNA Databases (NDNADs) (Hares, 2012, 2015; Karantzali et al., 2019). A variety of commercial capillary electrophoresis (CE)-based autosomal STR amplification kits have been developed and used for human identification, kinship relationship, and mixture deconvolution (Gill et al., 2006; Gymrek, 2017; Al-Eitan et al., 2019). SNPs are the most common type of genetic variation among the human genome and are also the most useful for studying human evolutionary history over long time scales (Agrafioti and Stumpf, 2007). Indels occur less frequently in the genome than SNPs. Furthermore, indel markers are almost unavailable around the forensic commonly used STRs, such as the expanded CODIS core loci STR set and the Extended European Standard Set (ESS) (Welch et al., 2012; Tan et al., 2018). Based on these reasons, SNP-STRs combine the advantages of both SNPs and STRs, provide more information than DIP-SNPs and SNP-SNPs, have more candidates than DIP-STRs, and offer the possibility of gaining better insights into population genetic processes.

Massively parallel sequencing (MPS) technology can directly detect each strand’s allele combinations separately. However, the higher costs and lack of consistent nomenclature, reporting standards, and existing national DNA database infrastructure to support statistical calculations, impose practical challenges for the introduction of MPS for routine forensic procedures (Just and Irwin, 2018). Capillary electrophoresis (CE) platforms are still the most commonly used strategy in forensic laboratories.

Our previous research reported 11 SNP-STR markers based on a CE platform (Tan et al., 2018). Furthermore, in conjunction with a polymerase chain reaction (PCR) technique based on an amplification refractory mutation system (ARMS), SNP-STRs can be used to target minor DNA at an excess of 100-fold of major DNA, providing a powerful method to analyze unbalanced DNA mixtures. The successful detection of cell-free fetal DNA in the peripheral blood of pregnant women using this method in our previous study demonstrated the effectiveness of this method for forensic unbalanced DNA mixture analysis (Wang et al., 2017). However, the linked STRs of the 11 SNP-STRs are uncommon in the expanded CODIS core loci STR set, ESS, or other forensic commonly used STR kits.

In order to achieve maximum compatibility with existing DNA databases, and further explore the capacity of SNP-STR markers to help analyze unbalanced DNA mixtures, we aimed to develop further SNP-STR loci based on commonly used STR loci. Eighteen novel SNP-STRs based on the expanded CODIS core loci set and other commonly used STR kits were developed. Stable and sensitive SNP-STR multiplex PCR panels based on the CE platform were established. The capabilities of the SNP allele-specific ARMS primers of these loci were evaluated using simulated binary unbalanced mixtures. Forensic parameters were estimated based on a survey of 113 individuals from the southwest Chinese Han population. Furthermore, as the likelihood ratio (LR) has become the most commonly used method by forensic communities to determine the weight of evidence (Gill et al., 2006, 2012), a probabilistic framework for LR calculation for the SNP-STR profiling results of two-person mixtures was developed in this study. Finally, we report on the first use of these markers in a case study to show the advantages and limitations of SNP-STRs for forensic practice.

## Genetic Background

**FIGURE 1:**
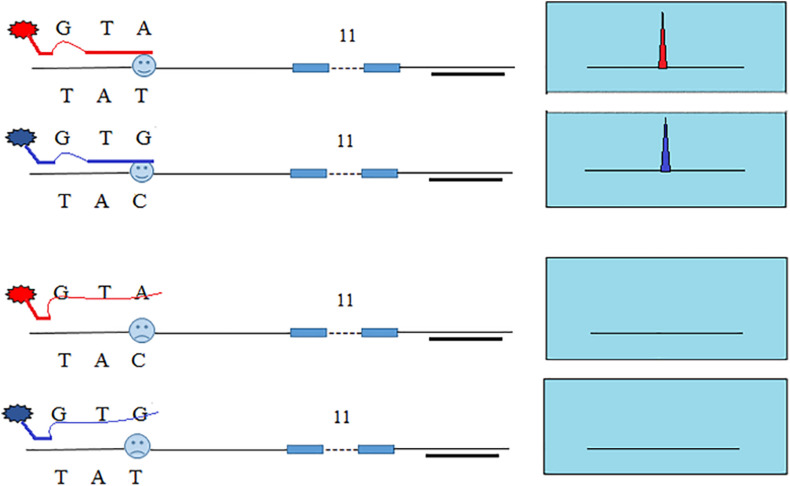
The principle of allele-specific primer design for SNP-STR markers using the ARMS technique. Each of the two allele-specific SNP primers is labeled with different fluorescent dyes. The common reverse primer is located downstream of the STR core region sequence. The obtained allele peaks are detected using capillary electrophoresis (CE), with the resultant product peaks containing two genotypes: (1) the color represents the SNP allele; (2) the size corresponds to the STR allele.

In our previous study, we developed SNP-STRs with ARMS technology to analyze unbalanced DNA mixtures (Wang et al., 2013, 2015). Figure 1 shows the design principle of the SNP-STR ARMS primers. The principle of this technique is to design two forward allele-specific primers that can target two different allele sequences of a SNP, respectively. SNP-STR ARMS primers require the 3′-end nucleotide of the two forward primers to be SNP allele-specific and the second or third nucleotide from the 3′-end to be changed to create a mismatch. The mismatch is introduced to ensure that the matched SNP-specific primer is refractory to PCR on the “mismatch” template (no amplification products obtained), but can still anneal to the complementary “match” template to complete PCR amplification (SNP allele-specific products obtained). A common reverse primer is located downstream of the STR core region sequence. Both SNP and STR genotypes can be obtained using a single PCR.

**FIGURE 2:**
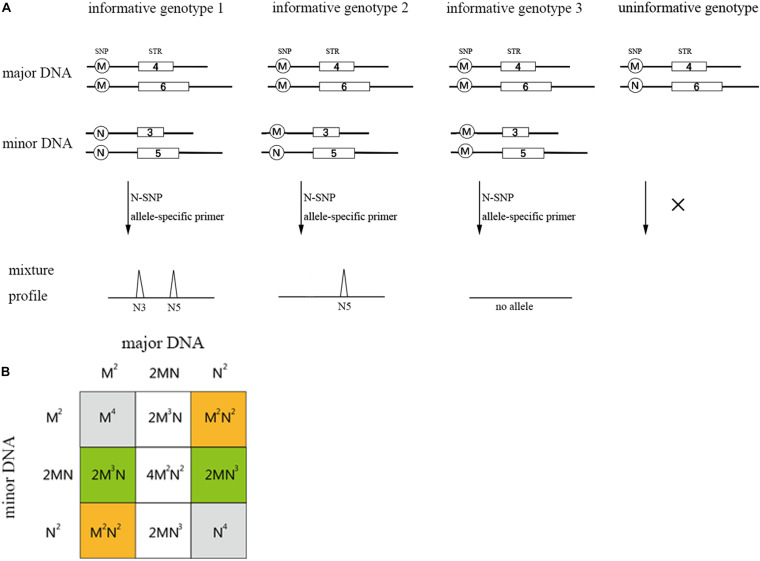
Possible genotype sets of the two contributors in a two-person mixture **(A)** and the probability table for all possible combinations **(B)**. The orange regions represent informative genotype 1 and the green regions represent informative genotype 2. Gray and blank represent informative genotype 3 and uninformative genotype, respectively. M and N in **(B)** represent the biallelic SNP allele frequency of an SNP-STR locus. The probability of informative genotypes for one SNP-STR locus is: *I* = M^2^N^2^ + M^2^N^2^ + 2M^3^N + 2MN^3^ = 2M^2^N^2^ + 2M^3^N + 2MN^3^.

Single nucleotide polymorphism-STR allele-specific primers allow for the selected amplification of the minor contributor’s genotype, as long as it has alleles that are absent in the major contributor’s genotype. For a two-person unbalanced mixture, the possible genotype configuration of the two contributors is represented by four scenarios (see Figure 2; Oldoni et al., 2015): (i) “informative genotype 1,” both the SNP genotypes of the major and minor DNA component are homozygous for the alternate allele, with a probability of M^2^N^2^ + M^2^N^2^ (M and N represent SNP allelic frequencies of a SNP-STR locus); (ii) “informative genotype 2,” the SNP genotype of the major DNA component is homozygous and the minor DNA component is heterozygous, with a probability of 2M^3^N + 2MN^3^. (iii) “informative genotype 3” represents the configuration when both the contributors are homozygous-MM, no alleles will be observed if we analyze the trace sample using the N-primer. The probability is M4 + N4; and (iv) when the SNP genotype of the major DNA component is heterozygous-MN, no specific SNP allele for the minor DNA component exists, which is called the “uninformative genotype.” The probability is 2M^3^N + 4M^2^N^2^ + 2MN^3^.

Under the situation of “informative genotype 1” and “informative genotype 2,” the SNP-STR allele-specific primer could be used to specifically target the minor DNA component from the mixture. Hence, the probability that the minor DNA component could be specifically targeted is M^2^N^2^ + M^2^N^2^ + 2M^3^N + 2MN^3^, which can be defined as the *I* value (probability of informative genotypes). This value can be used to assess the capability of the SNP-STR marker to target the minor DNA components in binary DNA mixtures that contain high background levels of major DNA components (Oldoni et al., 2015).

## Materials and Methods

### Ethics Statement

The participants provided written informed consent to participate in this study, and for participants under the age of 16 years, the legal guardian provided written informed consent to participate. No children under the age of 8 years participated in the study. All samples were obtained under the supervision of the Ethical Committee of Sichuan University (KS2019042).

Official permission to further analyze crime-related DNA samples and to present the corresponding data was obtained by the HI-TECH Industrial Sub-branch of Chengdu Municipal Public Security Bureau, Sichuan, China.

### Samples and DNA Preparation

Peripheral blood samples were collected from 113 unrelated Chinese Han individuals in Chengdu, China. Casework DNA samples from a sexual assault were used to report the first application of SNP-STR markers. A vaginal swab of the victim (No. 831-9) and reference blood samples of the victim (No. 831-1) and suspect (No. 831-2) were collected by the HI-TECH Industrial Sub-branch of Chengdu Municipal Public Security Bureau, Sichuan, China.

Genomic DNA was isolated using a whole blood extraction kit (BioTeke, Beijing, China) according to the manufacturer’s instructions and quantified spectrophotometrically using a NanoDrop 1000 Spectrophotometer (Thermo Fisher Scientific, Waltham, MA, United States).

### SNP-STR Candidate Screening

Single nucleotide polymorphism-STR loci were selected based on commonly used worldwide STRs, including those from the expanded CODIS core loci set, as well as other STRs available in commercial kits, such as the GlobalFiler PCR Amplification Kit (Thermo Fisher Scientific) and AGCU Expressmarker 22 kit (AGCU ScienTech Incorporation, Wuxi, Jiangsu, China) (Hares, 2015; He et al., 2017). The screening criteria were as follows: (i) SNP minor allele frequency in Han Chinese in Beijing, China (CHB), and Japanese in Tokyo, Japan (JPT), or East Asia (EAS) populations higher than 0.02, namely *I* > 0.04; (ii) SNP located within 400 bp of STR region; and (iii) targeted amplicons shorter than 550 bp.

The STRs mentioned above were searched for using the University of California Santa Cruz (UCSC, Santa Cruz, CA, United States) Genome Browser. SNPs in the flanking regions of STRs were filtered by dense “Base Position” and pack “STS Markers” in “Mapping and Sequencing” and full “Common SNPs (147)” with MAFs between 0.02 and 0.5 in “Variation.” All other options were hidden. The selected SNPs were confirmed to meet the screening criteria using the dbSNP database (NCBI, NIH, Bethesda, MD, United States). The SNP with the largest MAF or located closest to the STR was used in the following steps when there was more than one SNP around the STR locus.

### Genotyping SNP-STRs

#### Primer Design and Multiplex Establishment

General forward primers with the 3′ end starting with the SNP and a reverse primer downstream of the STR for one SNP-STR locus were designed using the Primer3 web version 4.0.0^1^. Allele-specific primers were designed by introducing a deliberate mismatch at the antepenultimate or penultimate base at the 3’ end of the forward primers. Two allele-specific forward primers and one standard reverse primer were obtained for each SNP-STR locus.

The SNP-STR multiplex was established to determine genotypes based on amplicon length and fluorophore label for each locus. The concentration of each primer was adjusted according to the capillary electrophoretic profiles of the amplification products generated by the primer mix reactions.

#### PCR Conditions and Genotyping

Singleplex PCR amplifications were performed in 10 μL reactions containing 5 μL Multiplex PCR Mix (Qiagen, Hilden, Germany), 0.5 μL primer mix (one forward primer and one reverse primer, 3 μM of each), 3.5 μL nuclease-free water, and 1 μL genomic DNA (1 ng/μL). Thermocycling was performed in a Mastercycler Nexus Gradient (Eppendorf, Hamburg, Germany) under the following conditions: initial denaturation at 95°C for 15 min; 31 cycles of 30 s at 94°C, 90 s at 58°C, and 60 s at 72°C; followed by a final extension step of 30 min at 60°C. The reaction and cycling conditions for the multiplex PCR amplifications using the primer mix were the same as those used for singleplex amplifications.

The PCR products were separated and detected by capillary electrophoresis using a 3130XL Genetic Analyser (Applied Biosystems, Foster City, CA, United States) by adding 1 μL of product to 9 μL of a 40:1 mixture of HiDi GeneScan 600 LIZ Size Standard (Thermo Fisher Scientific). Electrophoretic conditions were 9 s at 3 kV for injection and 1,500 s at 15 kV for the run. The initial data were analyzed using GeneMapper ID-X v1.2 software (Thermo Fisher Scientific), with the peak height threshold for each fluorescent dye set at 50 RFU.

A total of 113 DNA samples were profiled using the primer mix for the SNP-STR multiplex. Amplification of the positive control was performed using 1 ng ‘9948-control’ DNA (Health Gene Technologies, Ningbo, China). The negative control was a no-template control (NTC) consisting of ddH_2_O.

### Specificity of Primers

Forward primers (data not show) for Sanger sequencing were based on SNP-STR allele-specific primers for amplification using the reverse primers and obtain products containing SNP and STR loci between reactive primers. Three samples with SNP genotypes that were homozygous for both alleles and one heterozygote for each SNP-STR locus were amplified using sequencing primers. The resultant PCR products were sequenced from both directions using the Sanger method (sequencing performed by Invitrogen, Shanghai, China) after being recycled from polyacrylamide gel electrophoresis-separated bands. STR genotypes in the SNP-STR multiplex for some of the tested samples were confirmed using commercial STR kits (AGCU Expressmarker 22 kit and GlobalFiler PCR Amplification Kit).

### Sensitivity of PCR Assays

DNA samples with heterozygous SNP allele calls for each SNP-STR were selected to assess the minimum amount of template required for each allele-specific primer to obtain a positive profile. DNA samples diluted to 0.1, 0.05, 0.025, 0.01, and 0.005 ng/μL were amplified using the singleplex PCR conditions described in section Genotyping SNP-STRs.

### Stutter Percentage

Peaks that were one repeat smaller or larger than the true allele (±0.5 bases) were considered stutter peaks. The stutter percentage of each primer was calculated by dividing the stutter peak height (n ± 1 repeat unit) by the associated allele peak height with 113 unrelated Chinese Han individuals. Peak height threshold of stutters was set to 20 RFU.

### Simulated Mixture Detection

To assess the capability of each allele-specific primer to target the minor DNA components in extremely unbalanced binary mixtures, mixtures with a series of different ratios (1:50, 1:100, 1:200, and 1:500) were made (the amount of minor DNA template was fixed at 0.05 ng). For all the simulated mixtures, the major contributor was homozygous for a given SNP, while the minor contributor was heterozygous. Some of the DNA mixtures were genotyped using commercial STR kits (GlobalFiler PCR Amplification Kit) for comparison.

### Casework Example

It is important to note that for SNP-STR analysis of the mixture, the only information needed from the main contributor’s DNA is its SNP-heterozygosity or homozygosity. Suppose a mixed trace sample was collected from the victim, DNA of the victim and the suspect was both available. For SNP-STR analyses under this scenario, the steps were as follows: (i) amplify the reference DNA of the victim and suspect using the SNP-STR multiplex; (ii) select informative markers for which the major DNA contributor is SNP homozygous. The primers specific to the opposite SNP alleles are then used to target its amplification from trace samples; and (iii) assess the LR value of the DNA results.

For the traditional method, autosomal STR profiles of the trace sample and reference samples were obtained using the AGCU Expressmarker 22 kit (AGCU ScienTech Incorporation, Wuxi, Jiangsu, China) according to the manufacturer’s instructions. The SNP-STR genotype of the reference DNA was tested through SNP-STR multiplex PCR amplifications. Detection of the selected informative markers from the trace sample was based on Singleplex PCR amplifications. The reaction and cycling conditions, as well as the electrophoretic condition and data analysis method were the same as described in section PCR Conditions and Genotyping. A threshold of 50 RFU was used for peak calling.

### Statistical Analysis

#### General Parameters

Allele frequencies of each SNP-STR locus and corresponding SNPs were calculated for all 113 individuals. Exact tests for Hardy-Weinberg Equilibrium (HWE) as well as for linkage disequilibrium between all pairs of markers in the multiplex were performed using ARLEQUIN statistical software v3.5 (Excoffier and Lischer, 2010). To evaluate the detection performance of SNP-STRs for minor DNA in binary mixtures, the probabilities of informative genotypes (*I*) were estimated, using the formula *I* = 2M^2^N^2^ + 2M^3^N + 2MN^3^ (Figure 2), in which M and N are the frequencies of the M and N alleles in the 113 individuals, respectively. The average *I* value for the SNP was calculated and used to estimate the probabilities that SNP-STR markers (at least 5 and 10 loci) would be informative based on the cumulative binomial distribution of 40 trials (markers). Forensic parameters of SNP-STR and STR loci were assessed by calculating the matching probability (MP) and power of discrimination (DP) using Powerstats v1.2 (Promega, Madison, WI, United States).

#### LR Evaluation for Casework

Likelihood ratio is an established method that evaluates two contrasting hypotheses (prosecution vs. defense) (Gill and Haned, 2013; Dørum et al., 2014; Benschop et al., 2015; Slooten, 2017). Cereda et al. built an object-oriented Bayesian network (OOBN) to perform LR computation for DIP-STRs, and a series of casework DNA samples has been reported using this model (Cereda et al., 2014; Oldoni et al., 2017). Similar to the DIP-STR, the principle of SNP-STR used for two-person mixtures is based on the selected amplification of the minor contributor’s genotype. In this study, we constructed a similar Bayesian network in a Python environment to obtain LRs for particular SNP-STR profiling results. More detailed descriptions of the model are contained in the Supplementary Material. This model was constructed and the calculations were performed using pgmpy, a Python library for working with probabilistic graphical models^2^.

Likelihood ratio values for autosomal STR typing results were calculated using EuroForMix (Bleka et al., 2016). Population allele frequencies published by He et al. and Xin et al. were used for calculations (He et al., 2017; Adnan et al., 2018). LRs for SNP-STR profiling results were calculated using the above-mentioned Bayesian model. A detailed description is provided in the Supplementary Material. The SNP-STR allele frequencies used for calculations were derived from population research of 113 unrelated individuals in this study.

## Results

### Information on SNP-STRs

Nineteen SNP-STRs were identified that met the screening criteria (Table 1). The *I* value of these loci, calculated using the MAFs in dbSNP, were in the range of 0.123–0.375. All SNPs of the 19 SNP-STRs were verified to be biallelic, according to the dbSNP database.

**TABLE 1:** Information about the 19 SNP-STR loci.

SNP-STR	Chr. Location	distance (bp)	SNP Allele	SNP MAF (CHB/CHB+JPT/EAS)	*I* value
rs11642858-D16S539	16q24.1	15	A/C	C = 0.4390	0.310
rs58390469-D2S441	2p14	107	A/C	A = 0.4417	0.373
rs2325399-D6S1043	6q15	145	C/G	G = 0.3837	0.361
rs2070018-FGA	4q28	260	C/T	C = 0.0568	0.177
rs25768-D5S818	5q23.2	12	A/G	G = 0.0889	0.230
rs9531308-D13S317	13q31.1	114	A/C	A = 0.4167	0.354
rs8031604-Penta E	15q26.2	279	G/T	T = 0.0341	0.123
rs4847015-D1S1656	1q42	5	C/T	T = 0.1333	0.268
rs7962284-D12S391	12p13.2	192	C/T	C = 0.3095	0.366
rs7275705-Penta D	21q22.3	302	C/G	G = 0.1833	0.322
rs7786079-D7S820	7q21.11	64	A/C	C = 0.0333	0.136
rs57346531-D8S1179	8q24.13	270	A/G	G = 0.3083	0.326
rs2246512-D10S1248	10q26.3	69	A/G	G = 0.3690	0.225
rs17077990-D3S1358	3p21.31	271	C/G	G = 0.2222	0.301
rs17651965-CSF1PO	5q33.1	271	C/G	C = 0.4583	0.352
rs6736691-D2S1338	2q35	34	A/C	A = 0.1125	0.272
rs13413321-TPOX	2p25.3	147	G/T	G = 0.4302	0.369
rs9362476-SE33	6q14	169	C/T	T = 0.2907	0.375
rs11063971-VWA	12p13.31	89	C/T	C = 0.2167	0.177

*Chr. Location: the location of the SNP-STR on the chromosome; distance: number of bases between SNP and STR core area; SNP Allele: allele of SNP in SNP-STR locus; SNP MAF: minor allele frequency of SNP in dbSNP; CHB: Han Chinese in Beijing, China; JPT: Japanese in Tokyo, Japan; EAS: East Asia; *I* value: probabilities of informative genotypes for SNP-STR calculated using SNP frequencies from MAF.*

### Primers and Multiplex Panels

Two SNP allele-specific forward primers with mismatches introduced using the ARMS technique and one reverse primer were obtained for each SNP-STR locus. All target amplicons were shorter than 522 bp. High amplification efficiency was achieved with primers designed for 18 of the 19 SNP-STRs meeting the screening criteria. The rs11063971-VWA locus was excluded due to poor amplification. Details are listed in Table 2.

**TABLE 2:** Primer list of 19 SNP-STRs.

SNP-STR	Primer name	Primer sequence	5′ Dye	Panel	Amplicon size (bp)
rs11642858-D16S539	R	GGCAGATCCCAAGCTCTTCCTC			111–159
	F-A	GCATGTATCTATCATCCATCTCT**a**T	FAM	A	
	F-C	GCATGTATCTATCATCCATCTCT**t**G	JOE	B	
rs58390469-D2S441	R	GCTAAGTGGCTGTGGTGTTA			187–223
	F-A	TGAAAGGAGTGCAAGAGAAG**c**TA	FAM	A	
	F-C	TGAAAGGAGTGCAAGAGAAG**c**TC	JOE	B	
rs2325399-D6S1043	R	GAGCCACTTCCCATAATAAATCCT			229–293
	F-C	AAGTACCCTAACAAGTAACTCATC**c**TC	FAM	A	
	F-G	AAGTACCCTAACAAGTAACTCA**g**G**c**TC	JOE	B	
rs2070018-FGA	R	CCAAAATAAAATTAGGCATATTTACAAGCTAG			365–521
	F-C	GCCTTCCTTTTCCCTCTACTC**c**G	FAM	A	
	F-T	GCCTTCCTTTTCCCTCTACTC**c**A	JOE	B	
rs25768-D5S818	R	AGCCACAGTTTACAACATTTGTATCT			114–162
	F-A	GGGTGATTTTCCTCTTTGGTATCCTT**c**T	FAM	B	
	F-G	GGGTGATTTTCCTCTTTGGTATCCTT**c**C	JOE	A	
rs9531308-D13S317	R	CTCTGGACTCTGACCCATCTAACG			207–255
	F-C	GTGGGGAAATTTGTACATTCATTAATATACA**g**G	FAM	B	
	F-A	GTGGGGAAATTTGTACATTCATTAATATACA**g**T	JOE	A	
rs8031604-Penta E	R	TTTGGGTTATTAATTGAGAAAACTCCTTAC			363–468
	F-T	GGGTACCAATAACAAGAAAATTGTG**t**A	JOE	A	
	F-G	GGGTACCAATAACAAGAAAATTGT**t**GC	FAM	B	
rs4847015-D1S1656	R	GAGAAATAGAATCACTAGGGAACC			112–160
	F-C	TGTGTTGCTCAAGGGTCAACT**c**TG	TAM	A	
	F-T	TGTGTTGCTCAAGGGTCAACTG**c**A	ROX	B	
rs7962284-D12S391	R	TCCATATCACTTGAGCTAATTCCTCT			310–354
	F-T	CACCACTGCACTCCAGT**t**T	TAM	A	
	F-C	CACCACTGCACTCC**t**GCG	ROX	B	
rs7275705-Penta D	R	GAGCAAGACACCATCTCAAGAAAG			370–454
	F-G	GGTTAAATATCTCTTCAAATCTTTTGC**a**C	TAM	A	
	F-C	GGTTAAATATCTCTTCAAATCTTTTG**t**CG	ROX	B	
rs7786079-D7S820	R	AAGGGTATGATAGAACACTTGTCATAG			150–194
	F-C	CCTCATTGACAGAATTGCACC**t**C	TAM	B	
	F-A	CCTCATTGACAGAATTGCACC**t**A	ROX	A	
rs57346531-D8S1179	R	TACCTATCCTGTAGATTATTTTCACTGTG			354–406
	F-A	GAGCATAACAGAGGCACTGA**a**A	TAM	B	
	F-G	GAGCATAACAGAGGCACTGA**a**G	ROX	A	
rs2246512-D10S1248	R	CATATTAATGAATTGAACAAATGAGTGAGT			154–198
	F-A	CCCACCCCTGGATATTATAATTAA**a**AT	FAM	C	
	F-G	CCCACCCCTGGATATTATAATTAAC**g**C	JOE	C	
rs17077990-D3S1358	R	CAGAGCAAGACCCTGTCTCAT			343–391
	F-C	CTCAGCTTCAGCCCATAC**a**C	FAM	C	
	F-G	CTCAGCTTCAGCCCATAC**a**G	JOE	C	
rs17651965-CSF1PO	R	TTGCTAACCACCCTGTGTCTCAG			396–440
	F-G	GCTCMCACTCCGATGAG**g**TG	FAM	C	
	F-C	GCTCMCACTCCGATGAG**g**TC	JOE	C	
rs6736691-D2S1338	R	GGAGGGAGCCAGTGGATTT			138–206
	F-C	CTGCAGGTGGCCCATAA**a**C	ROX	C	
	F-A	CTGCAGGTGGCCCATA**t**TA	TAM	C	
rs13413321-TPOX	R	GGCACAGAACAGGCACTTAGG			215–263
	F-G	GGGGAGGAACTGGGAAC**t**C	TAM	C	
	F-T	GGGGAGGAACTGGGAAC**g**A	ROX	C	
rs9362476-SE33	R	GTCATGCCATTGCACTCCAAT			336–482
	F-C	**g**GCTGGAGCAGTTGTC**t**AC**t**A	TAM	C	
	F-T	**g**GCTGGAGCAGTTGTCG**g**T**t**A	ROX	C	
rs11063971-VWA	R	GGACAGATGATAAATACATAGGATGGATGG			197–253
	F-A	AGTTCCCACCTTCCAGAAG**c**G			
	F-B	AGTTCCCACCTTCCAGAAG**c**A			

*R in “Primer name” column: shared reverse primer; F-A/G/C/T in “Primer name” column: SNP A/G/C/T allele specific forward primer; in “Primer sequence” column, the underlined capital letters are the bases of allele-specific primers matched to SNP, the bold lowercase letters are bases mismatched to original sequence; 5′ Dye: the fluorophore label at the 5′-end of SNP allele-specific primer; Panel: the SNP-STR multiplex panel designation of the SNP primer (A, B, or C); Amplicon size: length range for target amplicons.*

**FIGURE 3:**
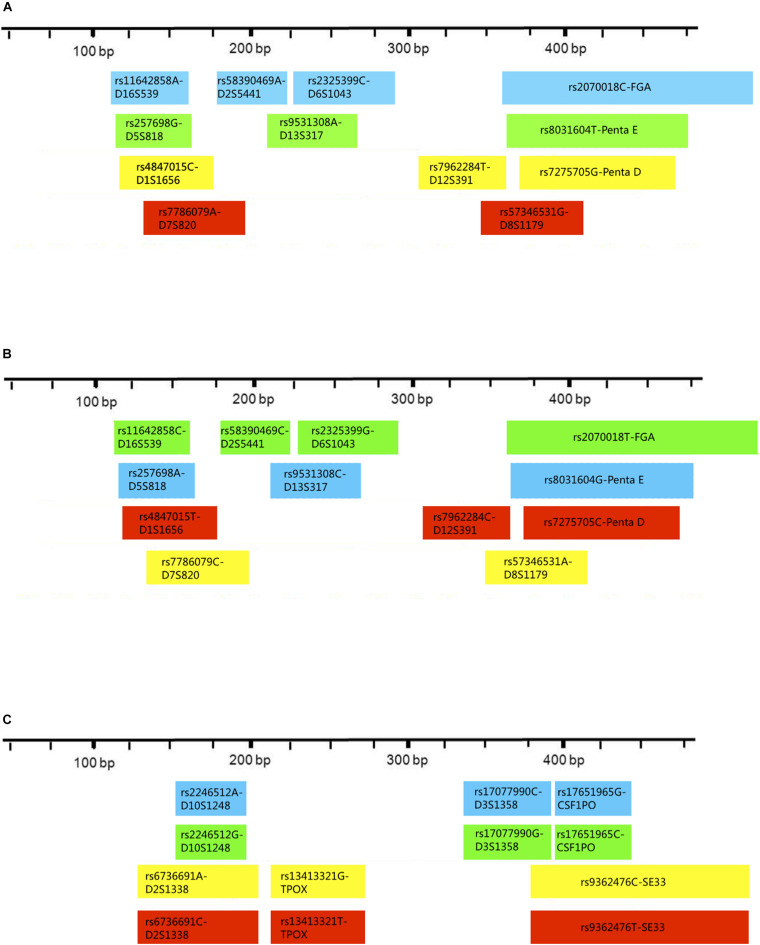
Distribution and fluorescent-dye colors of SNP allele-specific primers for 18 SNP-STRs in multiplex panels. The multiplexes consist of **Panel-A**, **Panel-B**, and **Panel-C**. Each block represents one pair of allele-specific forward and reverse primers of an SNP-STR locus, the width of each block indicates the length range of amplicons, and the color indicates peak color from the fluorophore label of each allele-specific primer in the profiles.

**FIGURE 4:**
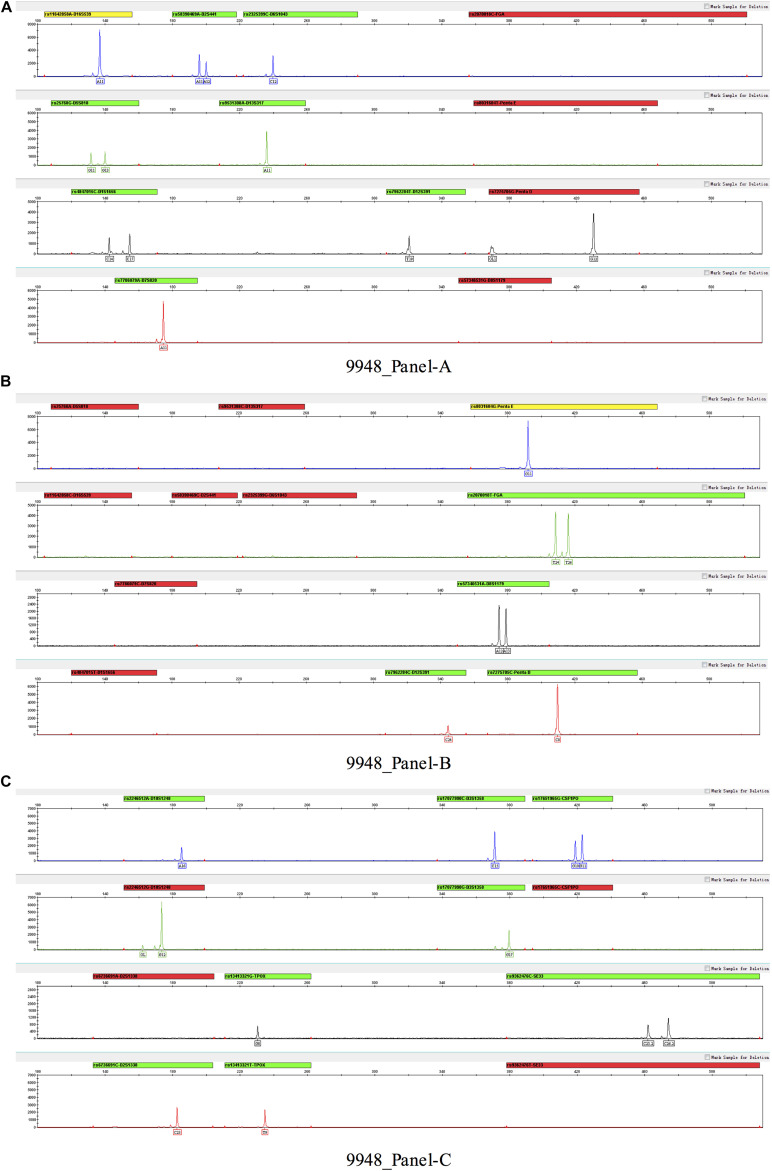
SNP-STR profiles of 9948-control DNA. **(A–C)** represent three panels (**Panel-A**, **Panel-B**, and **Panel-C**) respectively. No allelic peak and a red alert appear for one of the SNP allele-specific primers of a locus, indicating that the SNP genotype of this locus was homozygous for the alternate allele.

Three multiplex panels for 18 SNP-STRs were established: Panel-A, Panel-B, and Panel-C (Figure 3). The concentrations of each primer in the corresponding panels are listed in Supplementary Table 1. The SNP-STR profiles of 9948-control DNA are shown in Figure 4. Panel-A and Panel-B were composed of 12 identical SNP-STR loci with different allele-specific primers. Panel-C contained the remaining six loci.

### Specificity Tests

To examine the specificity of SNP-STR primers, samples were selected from the 113 individuals and analyzed using Sanger sequencing. The SNP and STR genotypes of these samples were concordant with the Sanger results. Supplementary Figure 1 shows the Sanger sequencing data for a sample with the genotypes A9/C9 at rs9531308-D13S317. The GlobalFiler PCR Amplification Kit (Thermo Fisher Scientific) plus the EX22 STR Kit, containing all of the 18 STRs in our SNP-STR system, were used to test the STR profiles for the selected samples. All of the STR profiling results were consistent with our SNP-STR multiplex results (data not shown).

### PCR Sensitivity

**FIGURE 5:**
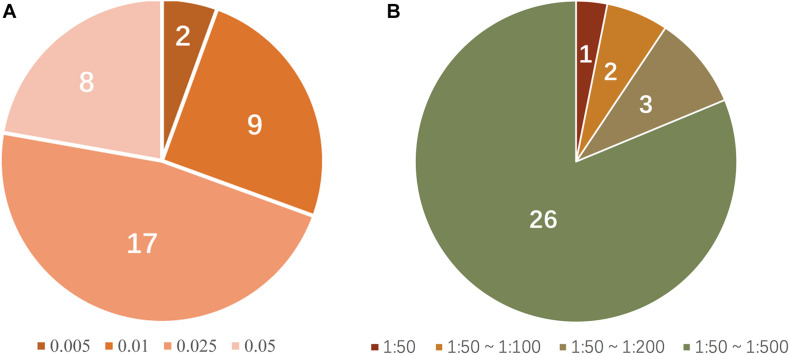
**(A)** Proportion of allele-specific primer sensitivity (peak height threshold: 50 RFU); numerals in the pie chart represent the number of primers matching the condition; colors represent the corresponding minimum amount of template required. **(B)** Proportion of mixture detection capacity of allele-specific primers (peak height threshold: 50 RFU); numerals in the pie chart represent the number of primers matching the condition; colors represent the range of the corresponding ratios of detectable mixtures.

Thirty-six allele-specific primers for the 18 SNP-STR loci were assessed. The primers for rs11642858C-D16S539 and rs17077990G-D3S1358 showed a positive result for amplification of DNA at the small amount of 0.005 ng. Primers for rs2070018C-FGA, rs25768A-D5S818, rs4847015T-D1S1656, rs7962284C-D12S391, rs7786079C-D7S820, rs2246512G-D10S1248, rs6736691A-D2S1338, rs58390469C-D2S441, and rs2325399G-D6S1043 required 0.01 ng DNA. Primers for rs9531308C-D13S317, rs7962284T-D12S391, rs7275705C-Penta D, rs7786079A-D7S820, rs57346531A-D8S1179, rs2246512A-D10S1248, rs6736691C-D2S1338, and rs9362476T-SE33 required at least 0.05 ng of template to generate a positive profile. The sensitivity of the remaining 17 primers reached 0.025 ng (Figure 5A). Supplementary Figure 2 shows examples of the sensitivity test results for the SNP-STR loci.

### Simulated Mixture Analysis

The simulated mixture tests required that the major DNA component was SNP homozygous for a given SNP-STR locus. Four primers (rs25768A-D5S818, rs8031604T-Penta E, rs7786079C-D7S820, and rs6736691A-D2S1338) were not tested because there were no homozygous genotypes for these SNPs according to the survey of 113 individuals. Among the 32 allele-specific primers tested, 26 successfully amplified the minor DNA without a masking effect from the major DNA in the mixtures at ratios from 1:50 to 1:500, three (rs4847015C-D1S1656, rs7962284T-D12S391, and rs2246512G-D10S1248) did so at ratios from 1:50 to 1:200, two (rs9362476C-SE33 and rs25768A-D5S818) did so at ratios from 1:50 to 1:100, and one (rs2070018T-FGA) did so at a ratio of 1:50 (Figure 5B). Supplementary Figure 3 shows examples of the detection performance in unbalanced DNA mixtures for SNP-STRs. Supplementary Figure 4 shows examples of simulated DNA mixtures analyzed using a conventional STR kit.

### Stutter Percentage

Owing to confusion with the minor contributor, a high percentage of stutter may make the analysis of the mixture more difficult. In this study, the stutter percentage was calculated by dividing the stutter peak height (n ± 1 repeat unit) by the associated allele peak height with 113 samples. The results of the average stutter percentage and SD for each locus are listed in Supplementary Table 2 and the average stutter plus three standard deviations were used to set the stutter filter threshold. The lowest average percentage of stutter was observed at locus rs58390469A-D2S441 (1.68%) and the highest was rs7962284C-D12S391 (9.16%). The recommended stutter filters would be useful for mixture analysis.

### SNP-STR Performance Assessment

Haplotype frequencies are listed in Supplementary Table S3. The locus with the minimum number of haplotypes was rs13413321-TPOX and that with the maximum was rs9362476-SE33. The SNP minor allele frequencies in the SNP-STRs ranged from 0.487 (rs13413321-TPOX) to 0.022 (rs7786079-D7S820) (Table 3). Observed heterozygosity, expected heterozygosity, and the probability values (*p*) of the HWE test are listed in Supplementary Table S4. The *p*-values for rs17651965-CSF1PO, rs6736691-D2S1338, and rs9362476-SE33 were all less than 0.05. There was no significant linkage disequilibrium among the SNP-STR combinations located on the same chromosome after Bonferroni correction (*p* < 0.0003).

**TABLE 3:** SNP information and the probabilities of informative genotypes (*I*) for each informative genotype combination in 113 Chinese Han individuals.

SNP-STR	SNP minor allele	Obs. Min-F	*I* of informative genotype 1	*I* of informative genotype 2	*I* of informative genotype 3
rs11642858-D16S539	C	0.447	0.122	0.25	0.133
rs58390469-D2S441	C	0.478	0.125	0.25	0.126
rs2325399-D6S1043	G	0.3982	0.115	0.25	0.156
rs2070018-FGA	C	0.097	0.015	0.144	0.665
rs25768-D5S818	A	0.062	0.007	0.103	0.774
rs9531308-D13S317	C	0.469	0.124	0.25	0.128
rs8031604-Penta E	T	0.058	0.006	0.097	0.787
rs4847015-D1S1656	T	0.128	0.025	0.173	0.578
rs7962284-D12S391	C	0.305	0.09	0.244	0.242
rs7275705-Penta D	G	0.2552	0.072	0.236	0.312
rs7786079-D7S820	C	0.022	0.001	0.041	0.915
rs57346531-D8S1179	G	0.3142	0.093	0.245	0.231
rs2246512-D10S1248	G	0.3142	0.093	0.245	0.231
rs17077990-D3S1358	G	0.19	0.047	0.213	0.432
rs17651965-CSF1PO	G	0.407	0.117	0.25	0.151
rs6736691-D2S1338	A	0.093	0.014	0.14	0.677
rs13413321-TPOX	G	0.487	0.125	0.25	0.126
rs9362476-SE33	T	0.385	0.112	0.249	0.165
average	−	−	0.0724	0.202	0.379
combined *I* value	−	−	0.747	0.984	0.999989

*Obs. Min-F: observed frequency of SNP minor allele; *I*: probability of informative genotypes for each SNP-STR calculated using observed frequency of SNP minor allele.*

The probabilities of informative genotypes (*I*) for each informative genotype combination, which were calculated using the observed SNP MAF to estimate the probability of detecting minor DNA in a mixture, are given in Table 3. On average, the current 18 SNP-STR markers demonstrated *I* values of 0.0724, 0.202, and 0.379 for three kind of informative genotype combinations, and a combined *I* value of 0.747, 0.984, and 0.999989, respectively, which means there is an approximate 74.7% probability of obtaining at least one informative genotype 1 marker of the minor contributor’s DNA in a two-person mixture using this set of SNP-STR loci, and 98.4 and 99.9989% for informative genotype 2 and informative genotype 3, respectively.

The results, shown in Table 4, indicate that if there are 40 SNP-STRs with comparable *I* value, 95.05% of DNA mixtures will have at least one informative genotype 1 marker, 92.83% will have at least five informative genotype 2 markers, and 97.06% will have at least ten informative genotype 3 markers based on the cumulative binomial distribution.

**TABLE 4:** Expected estimate for occurrence of informative markers using 40 SNP-STR markers.

Informative markers (Number)	Percentage of informative genotype 1 (*I* = 0.0724)	Percentage of informative genotype 2 (*I* = 0.202)	Percentage of informative genotype 3 (*I* = 0.379)
≥1	0.951	1	1
≥2	0.796	0.999	1
≥3	0.561	0.993	1
≥4	0.328	0.973	1
≥5	0.161	0.928	1
≥6	0.066	0.846	1
≥7	0.023	0.725	0.999
≥8	0.007	0.575	0.995
≥9	0.002	0.419	0.988
≥10	4.437E-04	0.279	0.971
≥11	9.183E-05	0.169	0.938

Forensic parameters containing match probability and power of discrimination of 18 SNP-STRs and 18 STRs are listed in Table 5. The match probability of the SNP-STRs ranged from 0.016 (rs9362476-SE33) to 0.185 (rs13413321-TPOX) and that of the STRs ranged from 0.016 (SE33) to 0.239 (TPOX). The power of discrimination of the SNP-STRs ranged from 0.815 (rs13413321-TPOX) to 0.984 (rs9362476-SE33) and that of the STRs ranged from 0.761 (TPOX) to 0.984 (SE33).

**TABLE 5:** Forensic parameters of SNP-STR and STR.

SNP-STR	MP	PD	STR	MP	PD
rs11642858-D16S539	0.061	0.939	D16S539	0.083	0.917
rs58390469-D2S441	0.044	0.956	D2S441	0.094	0.906
rs2325399-D6S1043	0.035	0.965	D6S1043	0.035	0.965
rs2070018-FGA	0.039	0.961	FGA	0.043	0.957
rs25768-D5S818	0.06	0.94	D5S818	0.072	0.928
rs9531308-D13S317	0.058	0.942	D13S317	0.078	0.922
rs8031604-Penta E	0.019	0.981	Penta E	0.019	0.981
rs4847015-D1S1656	0.064	0.936	D1S1656	0.064	0.936
rs7962284-D12S391	0.047	0.953	D12S391	0.064	0.936
rs7275705-Penta D	0.044	0.956	Penta D	0.063	0.937
rs7786079-D7S820	0.088	0.912	D7S820	0.096	0.904
rs57346531-D8S1179	0.042	0.958	D8S1179	0.058	0.942
rs2246512-D10S1248	0.042	0.958	D10S1248	0.092	0.908
rs17077990-D3S1358	0.071	0.929	D3S1358	0.126	0.874
rs17651965-CSF1PO	0.061	0.939	CSF1PO	0.127	0.873
rs6736691-D2S1338	0.045	0.955	D2S1338	0.045	0.955
rs13413321-TPOX	0.185	0.815	TPOX	0.239	0.761
rs9362476-SE33	0.016	0.984	SE33	0.016	0.984

*MP, match probability; PD, power of discrimination.*

### Profiling Results of the Casework

#### Traditional STR Profiling Results of the Casework

According to SWGDAM guidelines, if one or more loci have three or more alleles present, excluding tri-allelic loci, then the sample is assumed to be a mixture (SWGDAM, Accessed 6 November 2017). The autosomal STR profile of the trace sample had a maximum of four alleles at only one locus (vWA). Three loci (D3S1358, D2S1338, and D12S391) were shown to have three alleles, respectively. It can be inferred as a two-person mixture based on the maximum allele count (Dembinski et al., 2018). The STR profile of the trace sample showed that most alleles, even most of the alleles with a higher peak, correspond to the victim, indicating that the victim acts as the major component of this mixture. The combined LR of autosomal STR profiling results for the trace sample was approximately 2.86 × 10^3^.

**TABLE 6:** Autosomal STR profiling results of the victim, suspect, trace sample and resulting LR values.

STR loci	Victim (No. 831-1)		Suspect (No. 831-2)		Trace sample (No. 831-9)				LR
D3S1358	15	17	15	16	15 (367)^*a*^	16 (170)	17 (282)		1.568
D13S317	12		8	10	12 (353)				0.941
D7S820	11		8	12	8 (50)	11 (207)			3.092
D16S539	13		9	11	13 (396)				0.964
Penta E	11	16	13	16	11 (72)			11 (72)	0.836
D2S441	10	13	11	14	10 (81)	13 (72)		10 (81)	0.969
TPOX	8		8	11	8 (827)			8 (827)	0.853
TH01	7	9	7	9	7 (329)	9 (291)		7 (329)	1.263
D2S1338	17	23	19		17 (138)	19 (57)	23 (128)		5.654
CSF1PO	12	13	10	11	12 (80)	13 (66)		12 (80)	0.942
Penta D	9		8	12	9 (131)			9 (131)	0.983
D10S1248	14	15	13	15	14 (79)	15 (106)			1.127
D19S433	13	14	13	14	13 (65)			13 (65)	1.074
vWA	14	19	16	18	14 (150)	16 (56)	18 (65)	14 (150)	15.308
D21S11	30	32.2	29	31.2	30 (107)	32.2 (110)			0.893
D18S51	16	17	14	16	16 (174)	17 (80)		16 (174)	1.356
D6S1043	10	13	11	14	10 (56)			10 (56)	0.926
D8S1179	10		11	15	10 (129)				0.986
D5S818	10	11	10	11	10 (114)	11 (110)			1.244
D12S391	19	21	20		19 (88)	20 (57)	21 (80)		5.834
FGA	22	23	19	22.2	22 (83)	23 (75)			0.959
JointLR									2.86 × 10^3^

*^*a*^Allelic peak heights were shown in brackets.*

#### SNP-STR Profiling Results of the Casework

In this case study, the SNP genotypes of the victim and suspect constituted one locus of informative genotype 1, eight loci of informative genotype 2, six loci of informative genotype 3, and three loci of the uninformative genotype. For the trace sample, all informative alleles were successfully detected by using allele-specific primers targeting minor contributor’s alleles. The loci that belonged to informative genotype 3 showed no peaks, as expected. The SNP-STR alleles of the trace sample and suspect corresponded to all of these markers. The average and combined LR values of SNP-STR informative alleles for this casework were calculated using the Bayesian model mentioned previously, and the results are shown in Table 6. The combined LR was obtained by multiplication because the SNP-STR markers are assumed to be independent. The combined LR reached 7.14 × 10^7^.

**TABLE 7:** Informative genotype profiling results and LR values of the trace sample.

Combination	SNP-STR locus	Victim	Suspect	Trace sample	LR
Informative genotype 1	rs58390469-D2S441	C10-C13	A11-A14	A11, A14	14.195
Informative genotype 2	rs11642858-D16S539	A13-A13	A11-C9	C9	2.814
	rs2070018-FGA	T22-T23	C22.2-T19	C22.2	30.525
	rs9531308-D13S317	C12-C12	A8-C10	A8	2.451
	rs7275705-Penta D	C9-C9	G12-C8	G12	7.535
	rs57346531-D8S1179	A10-A10	G15-A11	G15	3.747
	rs17077990-D3S1358	C15-C17	C15-G16	G16	6.995
	rs17651965-CSF1PO	G12-G13	G11-C10	C10	4.470
	rs13413321-TPOX	G8-G8	G8-T11	T11	2.563
Informative genotype 3	rs2325399-D6S1043	C10-C13	C11-C14	NR	2.769
	rs25768-D5S818	G10-G11	G10-G11	NR	1.139
	rs8031604-Penta E	G11-G16	G13-G16	NR	1.129
	rs4847015-D1S1656	C11-C16	C11-C15	NR	1.318
	rs7786079-D7S820	A11-A11	A8-A11	NR	1.048
	rs2246512-D10S1248	A14-A15	A13-A15	NR	2.131
Uninformative genotype	rs7962284-D12S391	T19-C21	T20	−	−
	rs6736691-D2S1338	A17-C23	C19	−	−
	rs9362476-SE33	T17-C25.2	T17-C24.2	−	−

*Combination: possible genotype sets of the two contributors in a two-person mixture. Trace sample: profiling results of the trace sample (No. 831-9) by using allele-specific primers targeting informative alleles. NR represents no alleles was obtained.*

Profiling results of the trace sample using allele-specific primers targeting minor contributor’s alleles are shown in Supplementary Figure 6. All informative alleles were successfully detected. The loci that belonged to informative genotype 3 showed no peaks, as expected. The SNP-STR alleles of the trace sample and suspect corresponded to all of these markers.

The LRs of SNP-STR informative alleles for this casework were calculated using the Bayesian model mentioned above, and the results are shown in Table 7. The combined LR was obtained by multiplication because the SNP-STR markers are assumed to be independent. The average and combined LR values are shown in Table 8. The combined LR reached 7.14 × 10^7^.

**TABLE 8:** Average and combined LR for this casework.

Combination	Allele number	Average LR	Combined LR
Informative genotype 1	1	14.195	14.195
Informative genotype 2	8	7.638	4.76 × 10^5^
Informative genotype 3	6	1.589	10.481
Combined	15	−	7.14 × 10^7^

## Discussion

Our laboratory has previously reported 11 SNP-STRs, but few of the linked STRs are derived from commonly used databases (Tan et al., 2018). The discriminatory power for mixture analysis requires more loci for which more informative markers may be obtained. In this study, we aimed to develop more SNP-STR loci based on commonly used STRs, establishing a connection between SNP-STR markers and existing STR databases. Eighteen SNP-STR loci were screened here, of which 14 were derived from the expanded CODIS core loci set. Three loci (D6S1043, Penta D, and Penta E) are available in the AGCU Expressmarker 22 kit. We also included the most informative tetranucleotide loci studied to date, SE33, which is included in various CE-based kits (GlobalFiler, NGM Select, ESSPlex, PowerPlex ESI/ESX 17). SE33 is a core locus for the German National DNA Database (DAD) and has also been adopted by other laboratories in Europe (Butler, 2012).

As described above, the capability of an SNP-STR assay to target the minor DNA component of a binary DNA mixture with high background levels of the major DNA component can be assessed using the *I* value, which is based on the minor allele frequency of the SNP (Tan et al., 2018). Since the SNP-STRs we screened were limited to commonly used STRs, we set the minimum SNP MAF filter to 0.02 to obtain more loci. There are far fewer indels than SNPs across the genome. During screening of SNP-STR candidates, we found that almost no indel loci were located near these STR loci. DIP-STR loci with *I* > 0.04, based on the 24 commonly used STRs, cannot be accessed.

The purpose of establishing a multiplex system is to facilitate the investigation of population genetics and its application to forensic casework. Due to limitations in the number of loci and the length of the target amplicons, all primers couldn’t be mixed into a single panel. All 36 allele-specific SNP primers of the 18 SNP-STRs were eventually combined into three panels. Compared with singleplex profiling, the analytical efficiency was still greatly improved. Two sets of primers for each SNP-STR locus must be considered to obtain each sample’s genotype, increasing the complexity of data analysis. Furthermore, manual data handling might introduce errors. The development of integrated software for SNP-STR genotyping data, as a follow-up study, would greatly simplify the analysis and improve its overall efficiency and accuracy.

No differences were found between the Sanger sequencing and SNP-STR profiling results. Conventional STR kits were used to compare the STR genotypes of DNA samples obtained by the SNP-STR primers, and no differences were found. These results highlight the specificity of the SNP-STR multiplex system established in this study.

For the sensitivity analysis, all SNP-STR primers in this study successfully amplified DNA using 0.05 ng of template per reaction. The sensitivity was equivalent to that of DIP-STR assays (minimum detection limit was 0.03–0.1 ng). DIP-STRs also enable the specific detection of minor DNA even when the amount of major DNA is 1,000-fold higher (Castella et al., 2013; Oldoni et al., 2015). However, as the results of the simulated-mixtures test showed, compared with the allele-specific primers of DIP-STR markers, ARMS primers of SNP-STR markers with 1–3 consecutive or discontinuous base mismatches may not be able to achieve similar specificity due to interference from major DNA. Furthermore, mutual interference among primers exists in the multiplex. Singleplex SNP-STR primers are recommended for targeting informative alleles from mixtures.

The number of alleles at each locus was increased by the combination of STRs and SNPs and unrelated to the MAF of the SNP. According to a population survey of 113 individuals, there were 10 rs7786079-D7S820 haplotypes, which is an increase of four compared to the six alleles of D7S820. The MAF of rs7786079 was 0.079. While the MAF of rs58390469 was 0.4599, there were 11 rs58390469-D2S441 haplotypes, which is an increase of only three compared to the eight alleles of D2S441. The incorporation of SNPs linked to STRs increased the forensic performance. The cumulative match probability (CMP) and the cumulative power of discrimination (CPD) of the 18 SNP-STRs were 2.87 × 10^–24^ and >0.999999999999999, respectively, while the CMP and CPD of the 18 STRs were 4.56 × 10^–22^ and >0.999999999999999, respectively. In the HWE test, *p* values for three of the SNP-STR loci were less than 0.05, which may have been caused by the selection of samples from a relatively small group.

Given that the SNP-STR loci in this study were screened only with commonly used STRs, their average *I* value was lower than that of the DIP-STR loci selected from across the genome (0.33) (Tan et al., 2017). When the panel of SNP-STR markers was increased to 40 loci, it resulted in at least 15 informative markers with more than 95% probability, which significantly raised the probability of resolving a mixture and provides more compelling evidence for personal identification. Based on the average LR in Table 8, we can infer that if there are 40 SNP-STR markers, we would have a >95% probability of generating a LR value of 4.98 × 10^6^. Further, if there are 50 SNP-STR markers, we would have a >98% probability of generating a LR value of 4.27 × 10^6^. Therefore, more SNP-STR loci is imperative to increasing the individual discrimination ability of the SNP-STR panel. A combination of SNP-STR and DIP-STR markers might be interesting for future studies.

Y-STR is commonly used in the analysis of forensic mixtures, especially in male-female mixtures from sexual assault cases. Given paternal inheritance, Y-STR haplotypes can only be used to exclude unrelated males and cannot be used for personal identification (Gusmão et al., 2006). Some studies have reported the use of massively parallel sequencing, whereby both STR core sequence and flanking sequence can be obtained at the same time. However, studies have also found that the presence of STR stutters in the analysis of high-proportion DNA mixtures makes it difficult to distinguish the allelic sequence of minor components from the overall sequencing results (Parson et al., 2016; van der Gaag et al., 2016; Jäger et al., 2017). Our SNP-STR analysis using the ARMS technique for simulated high proportion mixtures did not encounter the same difficulty.

The successful detection of SNP-STR alleles in unbalanced two-person mixtures depends on how the SNP genotypes of the mixture contributors compare to each other. Distinct allelic configurations are observed for each marker in practice. The evaluation processes may become tedious if this needs to be done manually. The use of the Bayesian model could make the process easier. It can also help to make evaluative procedures less prone to possible errors. OOBN, developed for DIP-STR analysis by Cereda et al., has the advantage of being able to deal with cases where the genetic information of further individuals (other than the suspect) needs to be considered (typically when the suspect’s genotype is not available) (Cereda et al., 2014; Oldoni et al., 2017). However, neither can handle situations where extra minor alleles are observed, which suggests the presence of extra unknown contributors in the mixture. Future work should be conducted to address these gaps for both DIP-STRs and SNP-STRs.

We also report our first experience of the use of SNP-STR markers in casework. As expected, the highest average LR belonged to informative genotype 1. However, the locus (rs2070018-FGA), which belonged to informative genotype 2, had the highest LR (30.525). This is understandable because the allele frequency of C22.2 of rs2070018-FGA (0.018) is much smaller than those of A11 and A14 of rs58390469-D2S441 (0.199 and 0.177, respectively). An LR > 1 could be obtained even when no alleles were detected for the trace sample when the two contributors were both homozygous SNPs of the same kind. The LR value can range from extremely strong support of the prosecution hypothesis (LR ≥ 1 × 10^6^) to extremely strong support of the defense hypothesis (LR ≤ 1 × 10^–6^), given the evidence (Martire et al., 2014). In this casework, both the traditional STR and SNP-STR profiling results of the trace sample support the preposition that the victim and the suspect are the two contributors to the mixture. Compared to 2.86 × 10^3^ for the traditional STR method, the combined LR reached 7.14 × 10^7^ using the SNP-STR method in this casework example. There are three primary limitations in STR analysis. Firstly, minor DNA can be masked by major DNA if they share the same STR allele. In this situation, the SNP-STR method can target minor DNA if it has an SNP allele opposite to that of the major DNA. Secondly, some alleles for minor contributors may be absent using the traditional STR method if it is heavily imbalanced mixtures. Thirdly, it is sometimes difficult to distinguish a true STR allele of minor DNA from the stutter peaks of major DNA, or other noise signals in unbalanced mixtures (Bieber et al., 2016). Our results demonstrated that most allele-specific SNP-STR primers can target minor DNA at an excess of major DNA up to 1:500 with little influence from major DNA’s signals. Therefore, SNP-STR markers may have a distinct advantage in unbalanced mixture analysis compared to STR markers.

## Conclusion

In this study, we developed a novel SNP-STR system based on a CE platform. All 18 SNP-STR loci used in this study were derived from commonly used STRs, and the corresponding STR genotypes could be matched to the existing STR database. The development of the SNP-STR multiplex system simplifies the analytical process. SNP-STR allele-specific primers designed using the ARMS technique can be used to target the minor components in unbalanced binary DNA mixtures with little influence of major DNA’s signals, suggesting that these markers may have a distinct advantage in unbalanced mixture analysis compared to STR markers. SNP-STRs can be used as an alternative profiling technique that usefully complements the broad range of approaches available to forensic practitioners.

## Data Availability Statement

The datasets generated for this study can be found in the Figshare https://doi.org/10.6084/m9.figshare.13311758.v1.

## Ethics Statement

Written informed consent was obtained from the individual(s) for the publication of any potentially identifiable images or data included in this article.

## Author Contributions

HJ, LW, WL, and LZ designed this study. HJ and LW wrote the manuscript. ML and YT conducted the sample collection. RZ and SQ conducted the experiment. JW and LaZ analyzed the results. All authors reviewed the manuscript.

## Conflict of Interest

The authors declare that the research was conducted in the absence of any commercial or financial relationships that could be construed as a potential conflict of interest.

## Footnotes

1
http://primer3.ut.ee

2
https://github.com/pgmpy/pgmpy
