# Corrigendum: A novel SNP-STR system based on a capillary electrophoresis platform

**DOI:** 10.3389/fgene.2022.1036011

**Published:** 2022-10-27

**Authors:** Hui Jian, Li Wang, Meili Lv, Yu Tan, Ranran Zhang, Shengqiu Qu, Jijun Wang, Lagabaiyila Zha, Lin Zhang, Weibo Liang

**Affiliations:** ^1^ Department of Forensic Genetics, West China School of Basic Medical Sciences and Forensic Medicine, Sichuan University, Chengdu, China; ^2^ Department of Obstetrics and Gynecology, West China Second University Hospital, Sichuan University, Chengdu, China; ^3^ Key Laboratory of Birth Defects and Related Diseases of Women and Children (Sichuan University), Ministry of Education, Chengdu, China; ^4^ Department of Immunology, West China School of Basic Medical Sciences and Forensic Medicine, Sichuan University, Chengdu, China; ^5^ HI-TECH Industrial Sub-Branch of Chengdu Municipal Public Security Bureau, Chengdu, China; ^6^ Department of Forensic Medicine, School of Basic Medical Sciences, Central South University, Changsha, China

**Keywords:** SNP-STR, microhaplotype, capillary electrophoresis, forensic genetics, unbalanced DNA mixtures, likelihood ratio (LR)

In the original article, there was an error. Individual STR data are identifiable and cannot be published, and as such, the following material containing STR profiling results has been removed: **Table 6**, **Table 7**, **Supplementary Figures S5, S6** and **Supplementary Table S3** as a result, the numbering of the figures and tables have been adjusted accordingly.

A correction has been made to **SNP-STR Performance Assessment**, **paragraph 1**. The corrected paragraph appears as follows:

“Haplotype frequencies are listed in **Supplementary Table S3**. The locus with the minimum number of haplotypes was rs13413321-TPOX and that with the maximum was rs9362476-SE33. The SNP minor allele frequencies in the SNP-STRs ranged from 0.487 (rs13413321-TPOX) to 0.022 (rs7786079-D7S820) (**Table 3**). Observed heterozygosity, expected heterozygosity, and the probability values (*p*) of the HWE test are listed in **Supplementary Table S4**. The *p*-values for rs17651965-CSF1PO, rs6736691-D2S1338, and rs9362476-SE33 were all less than 0.05. There was no significant linkage disequilibrium among the SNP-STR combinations located on the same chromosome after Bonferroni correction (*p* < 0.0003).”

A correction has been made to **Profiling Results of the Casework**, **Traditional STR Profiling Results of the Casework**, **paragraph 1**. The corrected paragraph appears as follows:

“According to SWGDAM guidelines, if one or more loci have three or more alleles present, excluding tri-allelic loci, then the sample is assumed to be a mixture (SWGDAM, Accessed 6 November 2017). The autosomal STR profile of the trace sample had a maximum of four alleles at only one locus (vWA). Three loci (D3S1358, D2S1338, and D12S391) were shown to have three alleles, respectively. It can be inferred as a two-person mixture based on the maximum allele count (Dembinski et al., 2018). The STR profile of the trace sample showed that most alleles, even most of the alleles with a higher peak, correspond to the victim, indicating that the victim acts as the major component of this mixture. The combined LR of autosomal STR profiling results for the trace sample was approximately 2.86 × 10^3^.”

A correction has been made to **Profiling Results of the Casework**, **SNP-STR Profiling Results of the Casework**, **paragraphs 1–3**. The corrected paragraph appears as follows:

“In this case study, the SNP genotypes of the victim and suspect constituted one locus of informative genotype 1, eight loci of informative genotype 2, six loci of informative genotype 3, and three loci of the uninformative genotype. For the trace sample, all informative alleles were successfully detected by using allele-specific primers targeting minor contributor’s alleles. The loci that belonged to informative genotype 3 showed no peaks, as expected. The SNP-STR alleles of the trace sample and suspect corresponded to all of these markers. The average and combined LR values of SNP-STR informative alleles for this casework were calculated using the Bayesian model mentioned previously, and the results are shown in **Table 6**. The combined LR was obtained by multiplication because the SNP-STR markers are assumed to be independent. The combined LR reached 7.14 × 10^7^.”

The authors apologize for this error and state that this does not change the scientific conclusions of the article in any way. The original article has been updated.

