# Supplementary material for: A Novel SNP-STR System Based on a Capillary Electrophoresis Platform
Source: Front Genet. 2021 Feb 5;12:636821. doi: 10.3389/fgene.2021.636821 (PMC7893108; doi:10.3389/fgene.2021.636821)
Supplement: Supplementary file 1 [file Data_Sheet_1.docx]

Supplementary Material

# Supplementary Data

The likelihood ratio (LR) is an established method that evaluates two contrasting hypotheses (prosecution vs. defence) (Champod et al., 2016). For a two-person mixture collected from the crime scene which the genotype of the victim and suspect are considered as known, the prosecution hypothesis (Hp) asserts that the mixture originated from the victim and the suspect. The defence hypothesis asserts that the mixture originated from the victim and one unknown contributor. The numerator evaluates the strength of the evidence (E) if the prosecution hypothesis (Hp) is true and the denominator evaluates the strength of the evidence if the defence hypothesis (Hd) is true. The likelihood ratio is formulated as follows:

$LR=\frac{\Pr\left( E | H_{p,} I \right)}{Pr\left( E | H_{d,} I \right)}$ (1)

In Cereda et al.(Cereda et al., 2014), according to Bayes’ theorem, Eq. (1) can be translated into the following way:

$\frac{\Pr\left( H_{p} | E, I \right)}{Pr\left( H_{d} | E, I \right)}=\frac{\Pr\left( H_{p} | I \right)}{Pr\left( H_{d} | I \right)}\frac{\Pr\left( E | H_{p,} I \right)}{Pr\left( E | H_{d,} I \right)}$ (2)

Here, the variable E refers to the SNP-STR typing results of the trace sample ($E_{stain}$), the genotype of the victim ($E_{victim}$) and suspect ($E_{suspect}$). The likelihood ratio (LR) can thus be written as follows:

$$LR=\frac{\Pr\left( E_{stain},E_{suspect},E_{victim} | H_{p},I \right)}{\Pr\left( E_{stain},E_{suspect},E_{victim} | H_{d}, I \right)}$$

$=\frac{\Pr\left( E_{stain} | H_{p}, E_{suspect},E_{victim}, I \right)}{\Pr\left( E_{stain} | H_{d},E_{suspect},E_{victim}, I \right)}\frac{\Pr\left( E_{suspect},E_{victim} | H_{p}, I \right)}{\Pr\left( E_{suspect},E_{victim} | H_{d}, I \right)}$

$=\frac{\Pr\left( E_{stain} | H_{p}, E_{suspect},E_{victim}, I \right)}{\Pr\left( E_{stain} | H_{d},E_{suspect},E_{victim}, I \right)}$ (3)

Eq (3) is obtained by invoking the assumption that the victim's and the suspect's genotype (represented by $E_{victim}$ and $E_{suspect}$) are considered as known. That is to say they do not depend on whether the suspect is or is not a contributor to the trace sample, given the background information *I*.

In view of the observation that, for marker rs11642858-D16S539 in casework of this study, the victim is A- homozygous (A13-A13), the suspect has the genotype A11–C9. The trace sample shows C9 when using C-primer for Marker rs11642858-D16S539 (shown in Table 7), which is compatible with the suspect’s genotype. Now is the question: What is the degree of support for the proposition according to which the suspect contributed to the trace sample?

Bayesian networks are a type of probabilistic graphical model that uses Bayesian inference for probability computations. It is made up of Nodes and directed Links between them. Nodes represent variables in the Bayesian sense. Each node has a conditional probability table (CPT) that gives the probability of each of its values given every possible combination of values for its parents (conditioning case). Cereda et al. has built an object-oriented Bayesian network (OOBN) to perform the LR computation for DIP–STR, and a series of casework DNA samples has been reported by using this model (Cereda et al., 2014; Oldoni et al., 2017). Similar to DIP-STR, the principle of SNP-STR used for two-person mixtures are based on the selected amplification of the minor contributor’s genotype. In this study, we constructed a similar Bayesian network based on python environment to obtain likelihood ratios for particular SNP–STR profiling results. The first step, we started by constructing a single pedigree to represent the relationships between the victim, suspect and real criminal. A pictorial representation of this pedigree was shown in Figure 5.


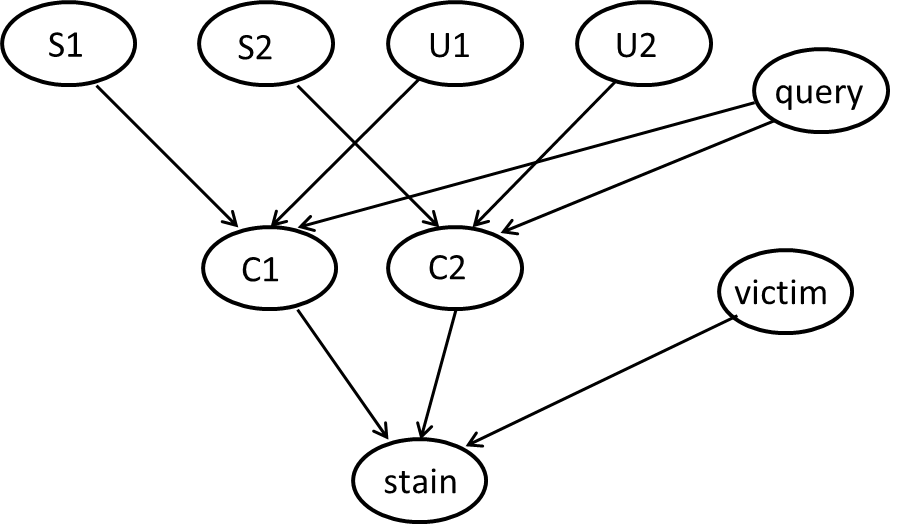


**Figure 5**. Pictorial representation of the Bayesian network to evaluate SNP–STR profiling results of two-person mixtures. The four nodes (S1, S2 and U1, U2) refer to the SNP-STR allelic constitution (on the two chromosomes) of the suspect and unknown contributor respectively. Here we defined the node victim refers to the victim’ SNP genotype. The node C1 and C2 represent the allelic constitution, on the two chromosomes, of the real criminal. The node query represents the alternative hypotheses, only have two states (true and false). If the node query is in the state true, that represents the prosecution hypothesis is true (the suspect is the real criminal), then the genotype of the real criminal should equal to the genotype of the suspect (C1=S1 and C2=S2). Otherwise it should be equal to the genotype of an unknown person from the relevant population (C1=U1 and C2=U2). The single node stain models the observed SNP–STR alleles in the trace sample by using allele-specific primers opposite to the SNP allele of the major contributor’s genotype. If both contributors are SNP-homozygous of different type (informative genotype 1), two SNP–STR alleles of the real criminal can be obtained if it is STR heterozygous (only one allele if the STR locus are homozygous too). If the victim is SNP-homozygous and the real criminal is SNP-heterozygous (informative genotype 2), then only the SNP–STR allele with the SNP allele opposite to that of the victim is revealed. If the two contributors are SNP-homozygous of the same type (informative genotype 3), no SNP–STR profiling results can be observed for the trace sample.

The four nodes (**S1/S2** and **U1/U2**) refer to the SNP-STR allelic constitution (on the two chromosomes) of the suspect and unknown contributor respectively. These nodes have twelve states (Aa, Ab, Ax, Ta, Tb, Tx, Ca, Cb, Cx, Ga, Gb, Gx) respectively. A/T/C/G represents the SNP allele(s). Letters a and b represent the STR alleles of the suspect, x represents the other STR alleles besides a and b that may appeared in the real criminal or unknown contributor. CPT of these nodes were shown in Table 9, contain the SNP-STR allelic frequencies obtained based on 113 individuals.

**Table 9.** Conditional probability table of the node **S1/S2/U1/U2**

| S1/S2/U1/U2 | P(vi) | S1/S2/U1/U2 | P(vi) |
| --- | --- | --- | --- |
| Aa | P(Aa) | Ca | P(Ca) |
| Ab | P(Ab) | Cb | P(Cb) |
| Ax | P(Ax) | Cx | P(Cx) |
| Ta | P(Ta) | Ga | P(Ga) |
| Tb | P(Tb) | Gb | P(Gb) |
| Tx | P(Tx) | Gx | P(Gx) |

As the name implies, the node **victim** refers to the victim. Only when the SNP genotype of the victim was homozygous, this SNP-STR locus can be defined as informative allele. This node has four states: Hom-A, Hom-T, Hom-C, Hom-G, represent the homozygous constitution of the victim, which can be obtained through SNP-STR typing in casework. For purely technical reasons, the CPT of this node is completed with equal probabilities (see Table 10).

**Table 10**. Conditional probability table of the node **victim**

| Victim | Hom-A | Hom-T | Hom-C | Hom-G |
| --- | --- | --- | --- | --- |
| P(vi) | 0.25 | 0.25 | 0.25 | 0.25 |

The node **query** represents the alternative hypotheses. This is a Boolean node, only have two states (*true* and *false*) (see Table 11). The node **C1** and **C2** represent the allelic constitution, on the two chromosomes, of the real criminal. Each of them has twelve states (Aa, Ab, Ax, Ta, Tb, Tx, Ca, Cb, Cx, Ga, Gb, Gx), as well as **S1**, **S2**, **U1** and **U2**. The state of **C1** depend on the state of the parent node **query, S1** and **U1**. **C2** depend on **query, S2** and **U2.** If the node **query** is in the state *true*, that represents the prosecution hypothesis is true (the suspect is the real criminal), then the genotype of the real criminal should equal to the genotype of the suspect (**C1**=**S1**, **C2**=**S2**). That is to say, if the node **S1** is in the state Aa, posterior probability 1 will be assigned to the state Aa of **C1**, 0 will be assigned to the other states (see Table 12). Otherwise it should be equal to the genotype of an unknown person from the relevant population (**C1**=**U1**, **C2**=**U2**), which means if the node **U1** is in the state Aa, posterior probability 1 will be assigned to the state Aa of **C1**, 0 will be assigned to the other states. For technical reasons, equal probabilities are assigned to the CPT of node **query** (shown in Table 11). The node **C1** and **C2** have an identical CPT, part of the CPT table was shown in Table 12.

**Table 11.** Conditional probability table of the node **query**

| query | true | False |
| --- | --- | --- |
| P(vi) | 0.5 | 0.5 |

**Table 12.** Part of the conditional probability table of the node **C1/C2**

| query | TRUE |  |  |  |  |  |  |  |  |  |  |  |  |  |  |  |  |  |  |  |  |  |  |  |  | … |
| --- | --- | --- | --- | --- | --- | --- | --- | --- | --- | --- | --- | --- | --- | --- | --- | --- | --- | --- | --- | --- | --- | --- | --- | --- | --- | --- |
| S1 | Aa |  |  |  |  |  |  |  |  |  |  |  |  | Ab |  |  |  |  |  |  |  |  |  |  |  | … |
| U1 | Aa | Ab | Ax | Ta | Tb | Tx | Ca | Cb | Cx | Ga | Gb | Gx |  | Aa | Ab | Ax | Ta | Tb | Tx | Ca | Cb | Cx | Ga | Gb | Gx | … |
| Aa | 1 | 1 | 1 | 1 | 1 | 1 | 1 | 1 | 1 | 1 | 1 | 1 |  | 0 | 0 | 0 | 0 | 0 | 0 | 0 | 0 | 0 | 0 | 0 | 0 | … |
| Ab | 0 | 0 | 0 | 0 | 0 | 0 | 0 | 0 | 0 | 0 | 0 | 0 |  | 1 | 1 | 1 | 1 | 1 | 1 | 1 | 1 | 1 | 1 | 1 | 1 | … |
| Ax | 0 | 0 | 0 | 0 | 0 | 0 | 0 | 0 | 0 | 0 | 0 | 0 |  | 0 | 0 | 0 | 0 | 0 | 0 | 0 | 0 | 0 | 0 | 0 | 0 | … |
| Ta | 0 | 0 | 0 | 0 | 0 | 0 | 0 | 0 | 0 | 0 | 0 | 0 |  | 0 | 0 | 0 | 0 | 0 | 0 | 0 | 0 | 0 | 0 | 0 | 0 | … |
| Tb | 0 | 0 | 0 | 0 | 0 | 0 | 0 | 0 | 0 | 0 | 0 | 0 |  | 0 | 0 | 0 | 0 | 0 | 0 | 0 | 0 | 0 | 0 | 0 | 0 | … |
| Tx | 0 | 0 | 0 | 0 | 0 | 0 | 0 | 0 | 0 | 0 | 0 | 0 |  | 0 | 0 | 0 | 0 | 0 | 0 | 0 | 0 | 0 | 0 | 0 | 0 | … |
| Ca | 0 | 0 | 0 | 0 | 0 | 0 | 0 | 0 | 0 | 0 | 0 | 0 |  | 0 | 0 | 0 | 0 | 0 | 0 | 0 | 0 | 0 | 0 | 0 | 0 | … |
| Cb | 0 | 0 | 0 | 0 | 0 | 0 | 0 | 0 | 0 | 0 | 0 | 0 |  | 0 | 0 | 0 | 0 | 0 | 0 | 0 | 0 | 0 | 0 | 0 | 0 | … |
| Cx | 0 | 0 | 0 | 0 | 0 | 0 | 0 | 0 | 0 | 0 | 0 | 0 |  | 0 | 0 | 0 | 0 | 0 | 0 | 0 | 0 | 0 | 0 | 0 | 0 | … |
| Ga | 0 | 0 | 0 | 0 | 0 | 0 | 0 | 0 | 0 | 0 | 0 | 0 |  | 0 | 0 | 0 | 0 | 0 | 0 | 0 | 0 | 0 | 0 | 0 | 0 | … |
| Gb | 0 | 0 | 0 | 0 | 0 | 0 | 0 | 0 | 0 | 0 | 0 | 0 |  | 0 | 0 | 0 | 0 | 0 | 0 | 0 | 0 | 0 | 0 | 0 | 0 | … |
| Gx | 0 | 0 | 0 | 0 | 0 | 0 | 0 | 0 | 0 | 0 | 0 | 0 |  | 0 | 0 | 0 | 0 | 0 | 0 | 0 | 0 | 0 | 0 | 0 | 0 | … |

The single node **stain** models the observed SNP–STR alleles in the trace sample by using allele-specific primers opposite to the SNP allele of the major contributor’s genotype. This node has fourteen states (Aa, Ab, Aab, Ta, Tb, Tab, Ca, Cb, Cab, Ga, Gb, Gab, X and NR). These states represent the SNP-STR alleles obtained from the trace sample. Aab, Tab, Cab and Gab represent the profiling results of trace sample when SNP genotype of the victim and real criminal are SNP-homozygous of different type. Aa, Ab, Ta, Tb, Ca, Cb, Ga and Gb represent the results when the victim is SNP-homozygous and the real criminal is SNP- heterozygous. The state NR represents a not observed peaks, that is a result obtained when the both contributors are SNP-homozygous of the same type. X is the sum of Ax、Tx、Cx and Gx in node **C1** and **C2**, reserved for results that show an allele corresponding to letter x.

Part of the CPT for the node **stain** was shown in Table 13. The state of the node **stain** depends on the state of the parent node **victim**, **C1** and **C2**, determines which STR alleles will appear in the trace sample. If both contributors are SNP-homozygous of different type (informative genotype 1), two SNP–STR alleles of the real criminal can be obtained if it is STR heterozygous (only one allele if the STR locus are homozygous too). If the victim is SNP-homozygous and the real criminal is SNP-heterozygous (informative genotype 2), then only the SNP–STR allele with the SNP allele opposite to that of the victim is revealed. If the two contributors are SNP-homozygous of the same type (informative genotype 3), no SNP–STR profiling results can be observed for the trace sample. In these cases, the node **stain** will assume the state NR. Posterior probability 1 will be assigned to the state NR, 0 will be assigned to the other states.

**Table 13**. Part of the CPT of node **stain**

| victim | | Hom-A | | |  | | |  | | |  | | |  | | |  | | |  | | |  | | |  | | |  | | |  | | |  | | |  | | |  | | |  | | |  | | |  | | |  | | |  | | |  | | |  | | |  | | |  | | |  | | |  | | |  | | |  | | |  | | |  | | |  | | |  | |  | | |  | | |  | | |  | | |  | | | … |
| --- | --- | --- | --- | --- | --- | --- | --- | --- | --- | --- | --- | --- | --- | --- | --- | --- | --- | --- | --- | --- | --- | --- | --- | --- | --- | --- | --- | --- | --- | --- | --- | --- | --- | --- | --- | --- | --- | --- | --- | --- | --- | --- | --- | --- | --- | --- | --- | --- | --- | --- | --- | --- | --- | --- | --- | --- | --- | --- | --- | --- | --- | --- | --- | --- | --- | --- | --- | --- | --- | --- | --- | --- | --- | --- | --- | --- | --- | --- | --- | --- | --- | --- | --- | --- | --- | --- | --- | --- | --- | --- | --- | --- | --- | --- | --- | --- | --- | --- | --- | --- | --- | --- | --- | --- | --- | --- | --- | --- | --- |
| C1 | | | Aa | | |  | | |  | | |  | | |  | | |  | | |  | | |  | | |  | | |  | | |  | | |  | | | Ab | | |  | | |  | | |  | | |  | | |  | | |  | | |  | | |  | | |  | | |  | | |  | | | Ax | | |  | |  | |  | | |  | | |  | | |  | |  | |  | | |  | | |  | | |  | | | … | | |
| C2 | Aa | | | Ab | | | Ax | | | Ta | | | Tb | | | Tx | | | Ca | | | Cb | | | Cx | | | Ga | | | Gb | | | Gx | | | Aa | | | Ab | | | Ax | | | Ta | | | Tb | | | Tx | | | Ca | | | Cb | | | Cx | | | Ga | | | Gb | | | Gx | | | Aa | | | Ab | | | Ax | | Ta | | | Tb | | | Tx | | | Ca | | | Cb | | | Cx | | | Ga | | | Gb | | | Gx | | | … | |
| Aa | 0 | | | 0 | | | 0 | | | 0 | | | 0 | | | 0 | | | 0 | | | 0 | | | 0 | | | 0 | | | 0 | | | 0 | | | 0 | | | 0 | | | 0 | | | 0 | | | 0 | | | 0 | | | 0 | | | 0 | | | 0 | | | 0 | | | 0 | | | 0 | | | 0 | | | 0 | | | 0 | | 0 | | | 0 | | | 0 | | | 0 | | | 0 | | | 0 | | | 0 | | | 0 | | | 0 | | | … | |
| Ab | 0 | | | 0 | | | 0 | | | 0 | | | 0 | | | 0 | | | 0 | | | 0 | | | 0 | | | 0 | | | 0 | | | 0 | | | 0 | | | 0 | | | 0 | | | 0 | | | 0 | | | 0 | | | 0 | | | 0 | | | 0 | | | 0 | | | 0 | | | 0 | | | 0 | | | 0 | | | 0 | | 0 | | | 0 | | | 0 | | | 0 | | | 0 | | | 0 | | | 0 | | | 0 | | | 0 | | | … | |
| Aab | 0 | | | 0 | | | 0 | | | 0 | | | 0 | | | 0 | | | 0 | | | 0 | | | 0 | | | 0 | | | 0 | | | 0 | | | 0 | | | 0 | | | 0 | | | 0 | | | 0 | | | 0 | | | 0 | | | 0 | | | 0 | | | 0 | | | 0 | | | 0 | | | 0 | | | 0 | | | 0 | | 0 | | | 0 | | | 0 | | | 0 | | | 0 | | | 0 | | | 0 | | | 0 | | | 0 | | | … | |
| Ta | 0 | | | 0 | | | 0 | | | 1 | | | 0 | | | 0 | | | 0 | | | 0 | | | 0 | | | 0 | | | 0 | | | 0 | | | 0 | | | 0 | | | 0 | | | 1 | | | 0 | | | 0 | | | 0 | | | 0 | | | 0 | | | 0 | | | 0 | | | 0 | | | 0 | | | 0 | | | 0 | | 1 | | | 0 | | | 0 | | | 0 | | | 0 | | | 0 | | | 0 | | | 0 | | | 0 | | | … | |
| Tb | 0 | | | 0 | | | 0 | | | 0 | | | 1 | | | 0 | | | 0 | | | 0 | | | 0 | | | 0 | | | 0 | | | 0 | | | 0 | | | 0 | | | 0 | | | 0 | | | 1 | | | 0 | | | 0 | | | 0 | | | 0 | | | 0 | | | 0 | | | 0 | | | 0 | | | 0 | | | 0 | | 0 | | | 1 | | | 0 | | | 0 | | | 0 | | | 0 | | | 0 | | | 0 | | | 0 | | | … | |
| Tab | 0 | | | 0 | | | 0 | | | 0 | | | 0 | | | 0 | | | 0 | | | 0 | | | 0 | | | 0 | | | 0 | | | 0 | | | 0 | | | 0 | | | 0 | | | 0 | | | 0 | | | 0 | | | 0 | | | 0 | | | 0 | | | 0 | | | 0 | | | 0 | | | 0 | | | 0 | | | 0 | | 0 | | | 0 | | | 0 | | | 0 | | | 0 | | | 0 | | | 0 | | | 0 | | | 0 | | | … | |
| Ca | 0 | | | 0 | | | 0 | | | 0 | | | 0 | | | 0 | | | 1 | | | 0 | | | 0 | | | 0 | | | 0 | | | 0 | | | 0 | | | 0 | | | 0 | | | 0 | | | 0 | | | 0 | | | 1 | | | 0 | | | 0 | | | 0 | | | 0 | | | 0 | | | 0 | | | 0 | | | 0 | | 0 | | | 0 | | | 0 | | | 1 | | | 0 | | | 0 | | | 0 | | | 0 | | | 0 | | | … | |
| Cb | 0 | | | 0 | | | 0 | | | 0 | | | 0 | | | 0 | | | 0 | | | 1 | | | 0 | | | 0 | | | 0 | | | 0 | | | 0 | | | 0 | | | 0 | | | 0 | | | 0 | | | 0 | | | 0 | | | 1 | | | 0 | | | 0 | | | 0 | | | 0 | | | 0 | | | 0 | | | 0 | | 0 | | | 0 | | | 0 | | | 0 | | | 1 | | | 0 | | | 0 | | | 0 | | | 0 | | | … | |
| Cab | 0 | | | 0 | | | 0 | | | 0 | | | 0 | | | 0 | | | 0 | | | 0 | | | 0 | | | 0 | | | 0 | | | 0 | | | 0 | | | 0 | | | 0 | | | 0 | | | 0 | | | 0 | | | 0 | | | 0 | | | 0 | | | 0 | | | 0 | | | 0 | | | 0 | | | 0 | | | 0 | | 0 | | | 0 | | | 0 | | | 0 | | | 0 | | | 0 | | | 0 | | | 0 | | | 0 | | | … | |
| Ga | 0 | | | 0 | | | 0 | | | 0 | | | 0 | | | 0 | | | 0 | | | 0 | | | 0 | | | 1 | | | 0 | | | 0 | | | 0 | | | 0 | | | 0 | | | 0 | | | 0 | | | 0 | | | 0 | | | 0 | | | 0 | | | 1 | | | 0 | | | 0 | | | 0 | | | 0 | | | 0 | | 0 | | | 0 | | | 0 | | | 0 | | | 0 | | | 0 | | | 1 | | | 0 | | | 0 | | | … | |
| Gb | 0 | | | 0 | | | 0 | | | 0 | | | 0 | | | 0 | | | 0 | | | 0 | | | 0 | | | 0 | | | 1 | | | 0 | | | 0 | | | 0 | | | 0 | | | 0 | | | 0 | | | 0 | | | 0 | | | 0 | | | 0 | | | 0 | | | 1 | | | 0 | | | 0 | | | 0 | | | 0 | | 0 | | | 0 | | | 0 | | | 0 | | | 0 | | | 0 | | | 0 | | | 1 | | | 0 | | | … | |
| Gab | 0 | | | 0 | | | 0 | | | 0 | | | 0 | | | 0 | | | 0 | | | 0 | | | 0 | | | 0 | | | 0 | | | 0 | | | 0 | | | 0 | | | 0 | | | 0 | | | 0 | | | 0 | | | 0 | | | 0 | | | 0 | | | 0 | | | 0 | | | 0 | | | 0 | | | 0 | | | 0 | | 0 | | | 0 | | | 0 | | | 0 | | | 0 | | | 0 | | | 0 | | | 0 | | | 0 | | | … | |
| X | 0 | | | 0 | | | 0 | | | 0 | | | 0 | | | 1 | | | 0 | | | 0 | | | 1 | | | 0 | | | 0 | | | 1 | | | 0 | | | 0 | | | 0 | | | 0 | | | 0 | | | 1 | | | 0 | | | 0 | | | 1 | | | 0 | | | 0 | | | 1 | | | 0 | | | 0 | | | 0 | | 0 | | | 0 | | | 1 | | | 0 | | | 0 | | | 1 | | | 0 | | | 0 | | | 1 | | | … | |
| NR | 1 | | | 1 | | | 1 | | | 0 | | | 0 | | | 0 | | | 0 | | | 0 | | | 0 | | | 0 | | | 0 | | | 0 | | | 1 | | | 1 | | | 1 | | | 0 | | | 0 | | | 0 | | | 0 | | | 0 | | | 0 | | | 0 | | | 0 | | | 0 | | | 1 | | | 1 | | | 1 | | 0 | | | 0 | | | 0 | | | 0 | | | 0 | | | 0 | | | 0 | | | 0 | | | 0 | | | … | |

note：Situations in which no alleles are revealed, due to low-template traces or other problems with the PCR process, are not taken into account in the paper.

The construction of this model and implementation of calculation were performed using pgmpy, a python library for working with probabilistic graphical models (<https://github.com/pgmpy/pgmpy>). Source code are freely available on request. We will introduce how to obtain LR value for this situation through the use of Bayesian model later.

For marker rs11642858-D16S539 in this casework, Eq (3) can be converted as follows:

$$LR=\frac{P\left( stain=C9 | query=true,victim=\mathrm{Hom}\_A,suspect=\left\{ A11,C9 \right\},I \right)}{P\left( stain=C9 | query=false,victim=\mathrm{Hom}\_A,suspect=\left\{ A11,C9 \right\},I \right)}$$

The value of the numerator means the probability of observing C9 from the trace sample – under the following conditioning ($query=true, victim=\mathrm{Hom}\_A,suspect=\left\{ A11,C9 \right\},I$). In turn, the denominator means the probability of observing C9 from the trace sample – under the following conditioning ($query=false, victim=\mathrm{Hom}\_A,suspect=\left\{ A11,C9 \right\},I$). The ratio between the two numbers thus found gives the likelihood ratio. Set *a*=11, *b*=9, *x* represents the sum of all the STR alleles other than 9 and 11 for marker rs11642858-D16S539.

On the basis of SNP-STR allelic frequency collected in this study (based on 113 individuals), the following prior probabilities of node S1/S2/U1/U2 were assigned:

P$\left\{ A11,A9,Ax,Ta,Tb,Tx,C11,C9,Cx,Ga,Gb,Gx \right\}$=P{0.204,0,0.341,0,0,0,0,0.261,0.19,0,0,0}.

The victim is A- homozygous, then the prior probabilities of node victim were assigned as: P{Hom-A，Hom-T，Hom-C，Hom-G}=P{1,0,0,0}.

To obtain value for the numerator, we need to set the state *true* to the node **query:** P{true, false}=P{1,0}. Then we can obtain the posterior probabilities of the state C9 of the node **stain** through the use of Bayesian network: P(C9) = 1.

To obtain value for the denominator, we need to set the state *false* to the node **query:** P{true, false}=P{0,1}. Then we can obtain the posterior probabilities of the state C9 of the node **stain** through the use of Bayesian network: P(C9) = 0.3554. The ratio between the two numbers, 2.814, gives the LR result of marker rs11642858-D16S539.

These values are easy to understand. For the numerator, if the suspect, whose genotype is A11–C9, is truly the real criminal, and the analyst analyses the trace sample using primers for the C-SNP, then it can reasonably be expected that C9 will be detected in the trace sample (assuming there were no disturbing or otherwise complicating factors). Posterior probability of 1 for C9 of node **stain** can thus be assigned. For the denominator, if the real criminal is an unknown contributor other than the suspect, and the C9 had been detected in the trace sample, the possible genotypes of the real criminal were as follows: C9 – C9, C9 – A11, C9 − Ax, for x ≠ 9, 11. The value of the denominator is thus given by the sum of the probabilities of these genotypes: ${(P_{C9})}^{2}+{2P}_{C9}P_{A11}+2P_{C9}P_{Ax}$=0.3554.

Here is an example for one informative locus. LR results of all informative markers for this casework were calculated by the same way. The results were shown in Table 7.

Cereda, G., Biedermann, A., Hall, D., and Taroni, F. (2014). Object-oriented Bayesian networks for evaluating DIP-STR profiling results from unbalanced DNA mixtures. *Forensic Sci Int Genet* 8(1)**,** 159-169. doi: 10.1016/j.fsigen.2013.09.001.

Champod, C., Biedermann, A., Vuille, J., Willis, S., and De Kinder, J. (2016). ENFSI Guideline for Evaluative Reporting in Forensic Science, A Primer for Legal Practitioners. *Criminal Law & Justice Weekly* 180**,** 189-193.

Oldoni, F., Castella, V., and Hall, D. (2017). Application of DIP-STRs to sexual/physical assault investigations: Eight case reports. *Forensic Science International Genetics* 30**,** 106.

# Supplementary Figures


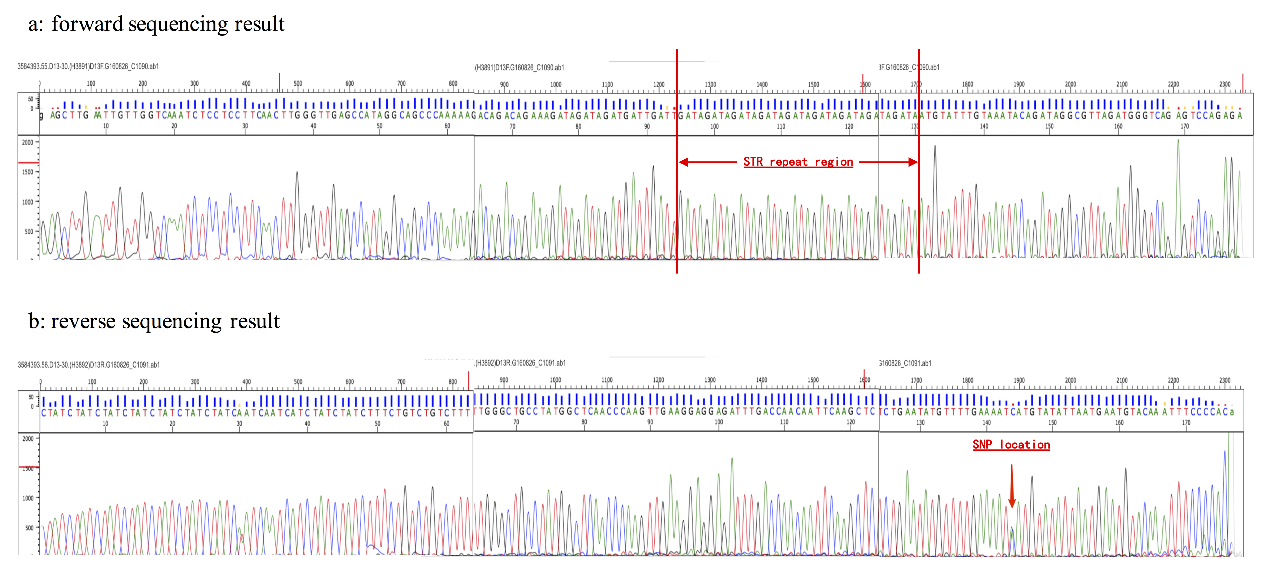


**Supplementary Figure S1.** Sanger sequencing data for sample with A9/C9 at rs9531308-D13S317.


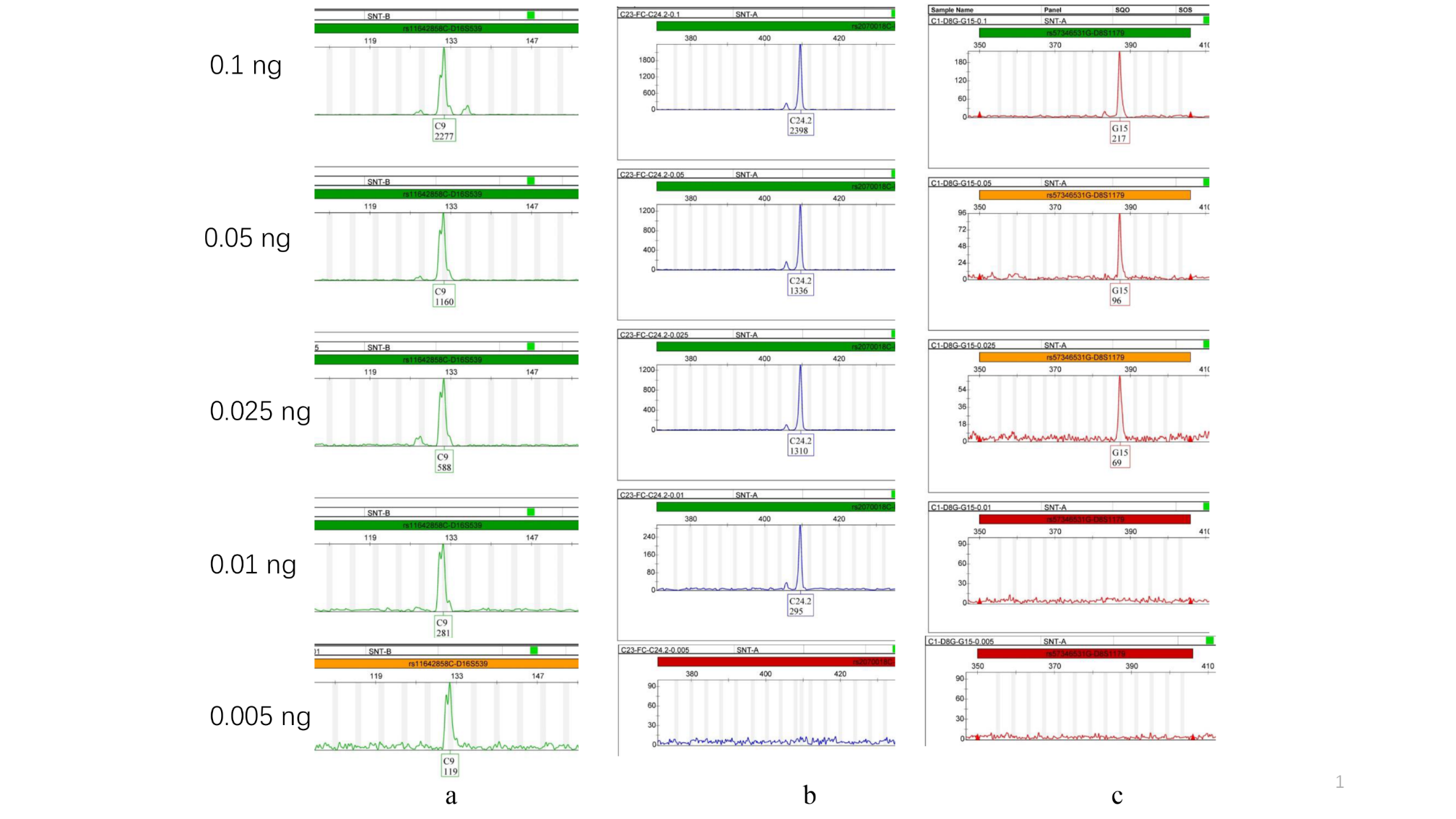


**Supplementary Figure S2.** Sensitivity results of rs11642858C-D16S539 (a), rs2070018C-FGA (b) and rs57346531G-D8S1179 (c). Amounts of DNA template amplified for each allele-specific primer from top to bottom were 0.1, 0.05, 0.025, 0.01 and 0.005 ng, respectively. Peak labels include allele name and height of allelic peak.


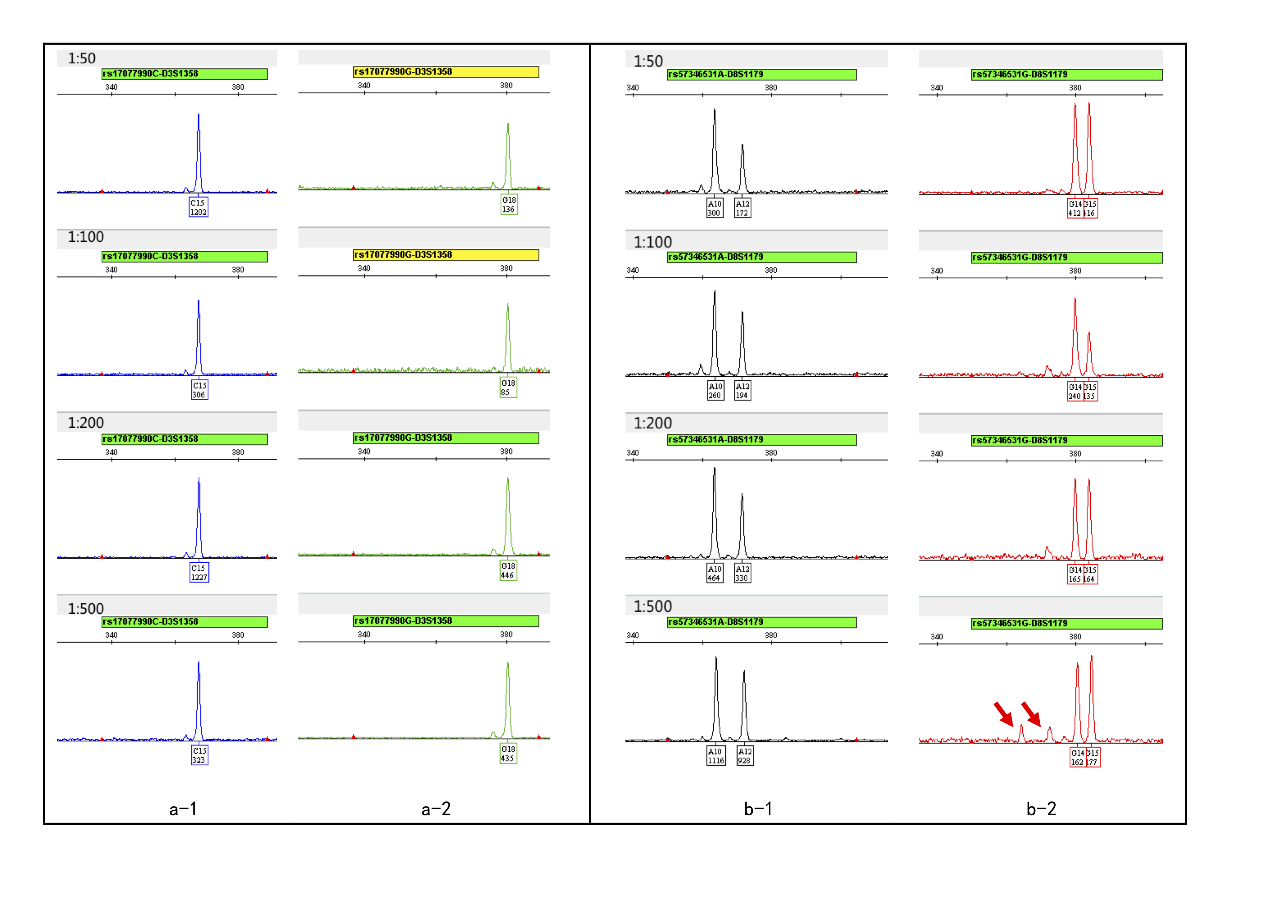


**Supplementary Figure S3.** Performance of rs17077990-D3S1358 in simulated binary unbalanced mixtures. Peak labels include allele name and height of allelic peak. Ratios of DNA mixture amplified for each allele-specific primer from top to bottom were 1:50, 1:100, 1:200, and 1:500, respectively. a-1: rs17077990C-D3S1358, genotypes of minor DNA and major DNA in simulated mixture were C15/G18 and G15/G17; a-2: rs17077990G-D3S1358, genotypes of minor DNA and major DNA in simulated mixture were C15/G18 and C14/C16.


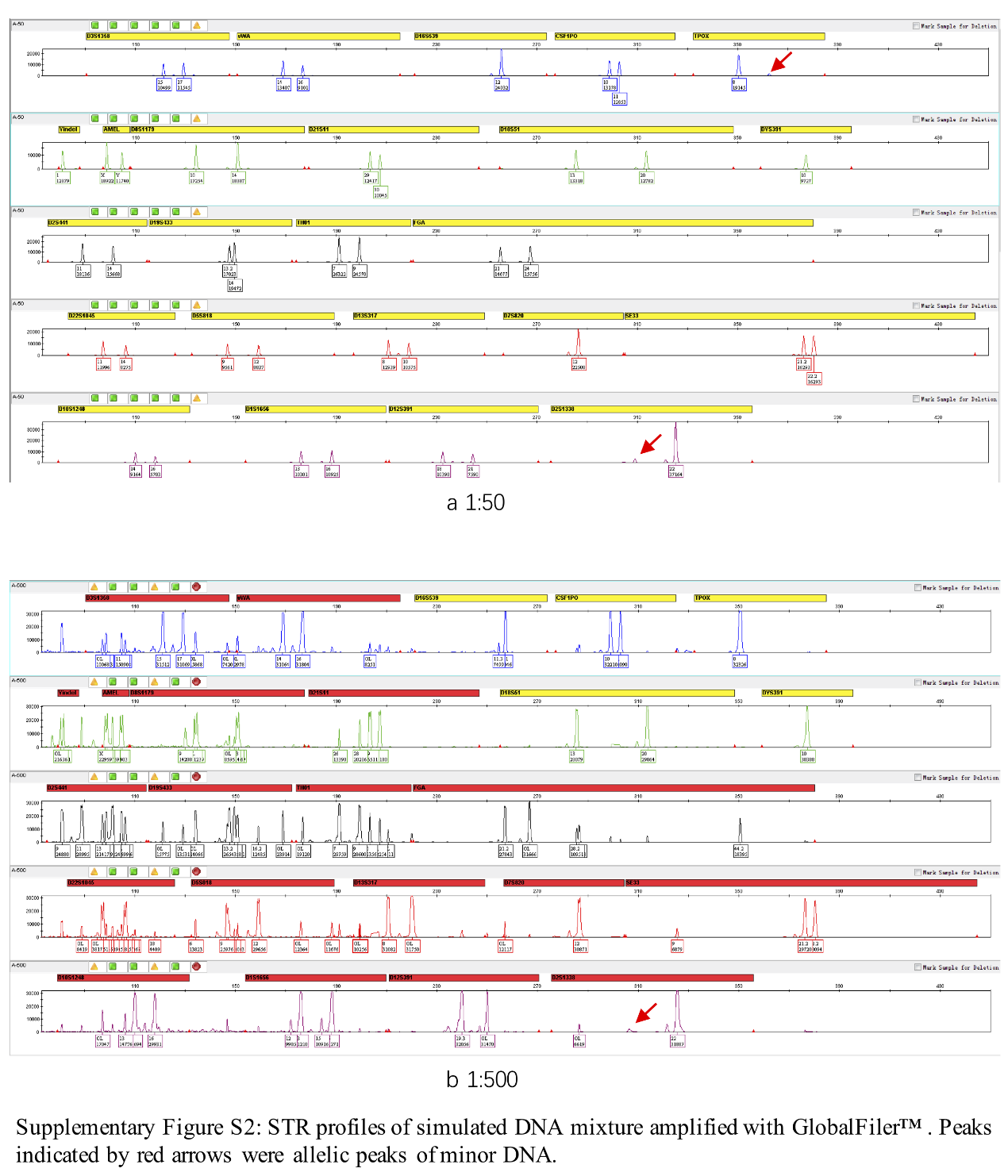


**Supplementary Figure S4.** STR profiles of simulated DNA mixtures amplified with GlobalFiler^TM^. Peaks marked with red arrows represent several alleles coming from minor DNA. Peak labels include allele name and height of allelic peak.
